# Converging peripheral blood microRNA profiles in Parkinson's disease and progressive supranuclear palsy

**DOI:** 10.1093/braincomms/fcae187

**Published:** 2024-05-31

**Authors:** Lukas Pavelka, Armin Rauschenberger, Ahmed Hemedan, Marek Ostaszewski, Enrico Glaab, Rejko Krüger, NCER-PD Consortium, Geeta Acharya, Gloria Aguayo, Myriam Alexandre, Muhammad Ali, Wim Ammerlann, Rudi Balling, Michele Bassis, Katy Beaumont, Regina Becker, Camille Bellora, Guy Berchem, Daniela Berg, Alexandre Bisdorff, Kathrin Brockmann, Jessica Calmes, Lorieza Castillo, Gessica Contesotto, Giuseppe Arena, Nico Diederich, Rene Dondelinger, Daniela Esteves, Guy Fagherazzi, Jean-Yves Ferrand, Manon Gantenbein, Thomas Gasser, Piotr Gawron, Soumyabrata Ghosh, Marijus Giraitis, Enrico Glaab, Clarissa Gomes, Elisa Gómez De Lope, Jérôme Graas, Mariella Graziano, Valentin Groues, Anne Grünewald, Wei Gu, Gaël Hammot, Anne-Marie Hanff, Linda Hansen, Maxime Hansen, Michael Heneka, Estelle Henry, Sylvia Herbrink, Sascha Herzinger, Michael Heymann, Michele Hu, Alexander Hundt, Ivana Paccoud, Nadine Jacoby, Jacek Jaroslaw Lebioda, Yohan Jaroz, Quentin Klopfenstein, Jochen Klucken, Rejko Krüger, Pauline Lambert, Zied Landoulsi, Roseline Lentz, Inga Liepelt, Robert Liszka, Laura Longhino, Victoria Lorentz, Paula Cristina Lupu, Clare Mackay, Walter Maetzler, Katrin Marcus, Guilherme Marques, Tainá Marques, Patricia Martins Conde, Patrick May, Deborah Mcintyre, Chouaib Mediouni, Francoise Meisch, Myriam Menster, Maura Minelli, Michel Mittelbronn, Brit Mollenhauer, Carlos Moreno, Friedrich Mühlschlegel, Romain Nati, Ulf Nehrbass, Sarah Nickels, Beatrice Nicolai, Jean-Paul Nicolay, Fozia Noor, Marek Ostaszewski, Sinthuja Paccontrolshek, Claire Pauly, Laure Pauly, Lukas Pavelka, Magali Perquin, Rosalina Ramos Lima, Armin Rauschenberger, Rajesh Rawal, Dheeraj Reddy Bobbili, Eduardo Rosales, Isabel Rosety, Kirsten Rump, Estelle Sandt, Stefano Sapienza, Venkata Satagopam, Margaux Schmitt, Sabine Schmitz, Reinhard Schneider, Jens Schwamborn, Jean-Edouard Schweitzer, Amir Sharify, Ekaterina Soboleva, Kate Sokolowska, Olivier Terwindt, Hermann Thien, Elodie Thiry, Rebecca Ting Jiin Loo, Christophe Trefois, Johanna Trouet, Olena Tsurkalenko, Michel Vaillant, Mesele Valenti, Sijmen Van Schagen, Liliana Vilas Boas, Maharshi Vyas, Richard Wade-Martins, Paul Wilmes, Evi Wollscheid-Lengeling, Gelani Zelimkhanov

**Affiliations:** Transversal Translational Medicine, Luxembourg Institute of Health (LIH), Strassen L-1445, Luxembourg; Parkinson’s Research Clinic, Centre Hospitalier de Luxembourg (CHL), Luxembourg L-1210, Luxembourg; Translational Neuroscience, Luxembourg Centre for Systems Biomedicine (LCSB), University of Luxembourg, Esch-sur-Alzette L-4367, Luxembourg; Biomedical Data Science Group, Luxembourg Centre for Systems Biomedicine (LCSB), University of Luxembourg, Esch-sur-Alzette L-4367, Luxembourg; Competence Centre for Methodology and Statistics, Translational Medicine Operations Hub, Luxembourg Institute of Health (LIH), Strassen L-1445, Luxembourg; Bioinformatics Core Unit, Luxembourg Centre for Systems Biomedicine (LCSB), University of Luxembourg, Esch-sur-Alzette L-4367, Luxembourg; Bioinformatics Core Unit, Luxembourg Centre for Systems Biomedicine (LCSB), University of Luxembourg, Esch-sur-Alzette L-4367, Luxembourg; Biomedical Data Science Group, Luxembourg Centre for Systems Biomedicine (LCSB), University of Luxembourg, Esch-sur-Alzette L-4367, Luxembourg; Transversal Translational Medicine, Luxembourg Institute of Health (LIH), Strassen L-1445, Luxembourg; Parkinson’s Research Clinic, Centre Hospitalier de Luxembourg (CHL), Luxembourg L-1210, Luxembourg; Translational Neuroscience, Luxembourg Centre for Systems Biomedicine (LCSB), University of Luxembourg, Esch-sur-Alzette L-4367, Luxembourg

**Keywords:** microRNA, Parkinson's disease, progressive supranuclear palsy, Boolean modelling, natural killer cell

## Abstract

MicroRNAs act via targeted suppression of messenger RNA translation in the DNA–RNA–protein axis. The dysregulation of microRNA(s) reflects the epigenetic changes affecting the cellular processes in multiple disorders. To understand the complex effect of dysregulated microRNAs linked to neurodegeneration, we performed a cross-sectional microRNA expression analysis in idiopathic Parkinson's disease (*n* = 367), progressive supranuclear palsy (*n* = 35) and healthy controls (*n* = 416) from the Luxembourg Parkinson's Study, followed by prediction modelling, enriched pathway analysis and target simulation of dysregulated microRNAs using probabilistic Boolean modelling. Forty-six microRNAs were identified to be dysregulated in Parkinson's disease versus controls and 16 in progressive supranuclear palsy versus controls with 4 overlapping significantly dysregulated microRNAs between the comparisons. Predictive power of microRNA subsets (including up to 100 microRNAs) was modest for differentiating Parkinson's disease or progressive supranuclear palsy from controls (maximal cross-validated area under the receiver operating characteristic curve 0.76 and 0.86, respectively) and low for progressive supranuclear palsy versus Parkinson's disease (maximal cross-validated area under the receiver operating characteristic curve 0.63). The enriched pathway analysis revealed natural killer cell pathway to be dysregulated in both, Parkinson's disease and progressive supranuclear palsy versus controls, indicating that the immune system might play an important role in both diseases. Probabilistic Boolean modelling of pathway dynamics affected by dysregulated microRNAs in Parkinson's disease and progressive supranuclear palsy revealed partially overlapping dysregulation in activity of the transcription factor EB, endoplasmic reticulum stress signalling, calcium signalling pathway, dopaminergic transcription and peroxisome proliferator-activated receptor gamma coactivator-1α activity, though involving different mechanisms. These findings indicated a partially convergent (sub)cellular end-point dysfunction at multiple levels in Parkinson's disease and progressive supranuclear palsy, but with distinctive underlying molecular mechanisms.

## Teaser

Pavelka *et al.* demonstrated convergent dysregulation in the natural killer cell signalling pathway in Parkinson's disease and progressive supranuclear palsy versus controls using microRNA target gene mapping. *In silico* modelling of dysregulated microRNA revealed partially convergent end-point cellular dysfunction involving shared pathways for both diseases with distinctive underlying molecular mechanisms.

## Graphical Abstract

**Graphical Abstract:**
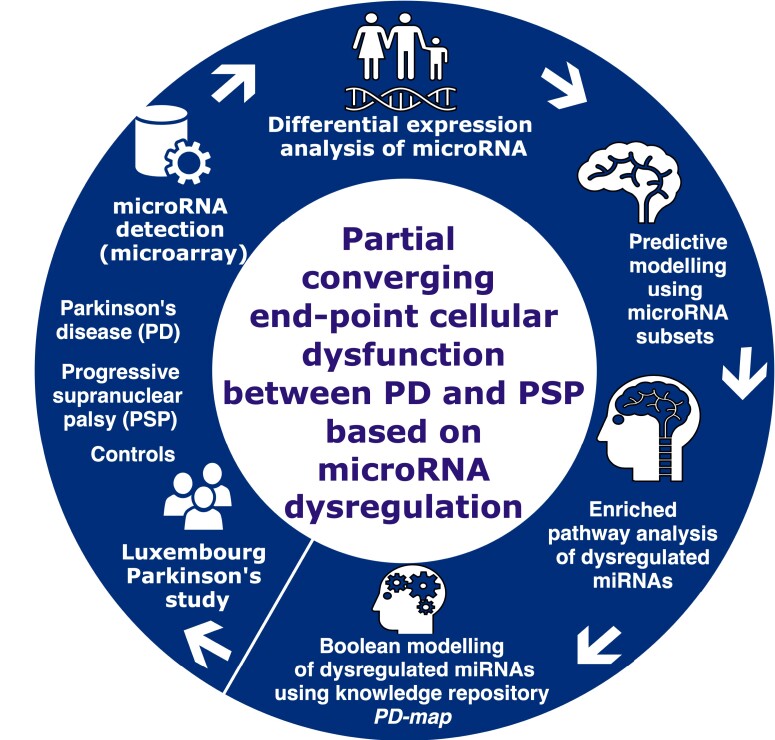

## Introduction

Parkinson's disease and progressive supranuclear palsy belong to a group of progressive neurodegenerative disorders with common parkinsonian motor features (bradykinesia, extrapyramidal rigidity, dystonia, dysphagia or freezing of gait) presenting a diagnostic dilemma for clinicians, especially early in the disease course.^1^ And yet, the prognosis and management of both disease groups differ substantially necessitating an early diagnostic biomarker for facilitation of individually tailored therapies as well as stratification for clinical trials on potential disease-modifying drugs. Whereas Parkinson’s disease is a prototypic α-synucleinopathy histopathologically defined by abnormal aggregates of α-synuclein (α-Syn) in the form of Lewy bodies (LBs) and Lewy neurites (LNs^2^), progressive supranuclear palsy is the most common form of atypical parkinsonism defined at autopsy by insoluble four-repeat isoform of cytoplasmic tau protein aggregates.^3^ However, histopathological classification of the neurodegenerative disorders has its challenges. A lesson learned from both monogenic cases and post-mortem studies in Parkinson’s disease is that neither does the absence of α-Syn inclusions necessarily exclude a clinical presentation of Parkinson’s disease (e.g. Parkinson’s disease due to certain *LRRK2* variants or *PRKN* mutations) nor the burden of these aggregates linearly corresponds to the quantitative cellular loss in the neurodegenerative disorders.^4,5^ Historically, the syndromic clustering across the neurodegenerative diseases has led to partly nosological entities that might eventually have common underlying molecular aetiology (e.g. Parkinson’s disease and dementia with LB; amyotrophic lateral sclerosis and frontotemporal dementia; progressive supranuclear palsy and corticobasal syndrome).^6^

On this point, several common pathways have been identified to be dysfunctional across different classes of neurodegenerative disorders including mitochondrial homeostasis,^7^ protein quality control and maintenance, maladaptive response of immune system, autophagy/lysosomal function and vesicle trafficking.^6^ In this context, research into shared genetic risk factors between Parkinson’s disease and other neurodegenerative disorders has identified multiple common genetic risk loci for Parkinson’s disease, dementia with LB, Alzheimer’s disease and progressive supranuclear palsy,^8-11^ further underpinning a possibility of converging molecular pathways across the neurodegenerative disorders. In addition to the coding mutations, epigenetic dysregulation has been suggested to play an important role in the pathophysiological cascades eventually leading to the progressive neurodegeneration.^12^

MicroRNAs (miRNAs) belong to a group of small non-coding single-stranded RNAs (sncRNAs) regulating the cellular pathways through the gene expression by interfering into the central DNA–messenger RNA (mRNA)–protein axis via mRNA suppression. The magnitude of miRNA effect is important to stress, as one miRNA is able to bind many mRNAs and one mRNA can be a target of multiple miRNAs, hence regulating the expression of hundreds of genes.^13,14^ Indeed, a growing body of evidence has emerged in favour of a substantial miRNA dysregulation across the neurodegenerative disorders including Alzheimer's disease,^15,16^ frontotemporal dementia,^17^ multiple system atrophy^18^ and Parkinson's disease.^19-25^ Such a dysregulation at the epigenetic level might provide an additional insight into the pathogenesis and progression of the neurodegenerative disorders.

Furthermore, miRNAs were suggested as potential diagnostic biomarkers due to the following: (i) their stability in the body fluids; (ii) without further structural modification; and (iii) are independent from circadian cycle. However, previous studies on the utility of miRNAs as a biomarker have been limited by low sample sizes of studied cohorts, by the variability in miRNA detection method, by a biofluid used and by different study set-ups, thus hampering the reproducibility of the results. Additionally, aging and sex play a substantial role in the miRNA expression levels leading to methodological difficulties when applied in the age-related disorders with disproportionate prevalence of males versus females such as seen in Parkinson’s disease or Alzheimer’s disease.^26^ Given these limitations, the overlap of significantly dysregulated miRNAs between studies has been extremely low and generally based on *ad hoc* comparison to previous reported studies on miRNAs. To our knowledge, only one large meta-analysis by Schulz *et al.*^27^ has systematically endeavoured to identify significantly dysregulated miRNAs in Parkinson’s disease in comparison to healthy individuals across multiple studies.

Furthermore, investigations into miRNA profiles in patients with atypical parkinsonism such as progressive supranuclear palsy have been scarce and limited by very low sample sizes in comparison to Parkinson’s disease.^28-32^ Moreover, the majority of previous studies lack a genetic screening for Parkinson’s disease–related genes in all compared groups, and yet the Parkinson’s disease–related mutation carriers were shown to present distinctive patterns of miRNA expression in comparison to idiopathic Parkinson’s disease.^33^

As miRNAs have a broad profile of activity, it is challenging to identify molecular mechanisms they regulate. One of the approaches is calculating enrichment of miRNA targets in known pathway databases, like Molecular Signatures Database (MSigDb^34^). To further narrow down their profile of activity, it is possible to use knowledge repositories focused on Parkinson’s disease mechanisms. One of such repositories is ‘Parkinson’s disease map’, a repository with over 100 pathways representing molecular neuropathology of Parkinson’s disease.^35^ Importantly, the contents of the Parkinson’s disease map are encoded in a format allowing their transformation into computational models that can be simulated using an approach called probabilistic Boolean modelling (PBM).^36^ PBM can be parameterized using miRNA expression profiles, providing an insight into dynamic of specific pathways and complementing the enrichment analysis.

In order to investigate these layers of pathway complexity, we integrated the genotyped sample of idiopathic Parkinson’s disease, controls and comparatively large sample of progressive supranuclear palsy patients with whole blood–derived miRNA microarray data from Luxembourg Parkinson's Study.^33^ Specifically, we performed all analysis after excluding all carriers of Parkinson’s disease–linked mutations identified via NeuroChip (Illumina) or by targeted re-sequencing of *GBA1* via PacBio.^37^

The primary objectives of this study were (i) to perform a cross-sectional analysis of the miRNA expression profile of idiopathic Parkinson’s disease, progressive supranuclear palsy and controls; (ii) to investigate the effects of age/sex on miRNA profiles; (iii) to fit prediction models to investigate potential use of miRNA expression signatures to use as diagnostic biomarker between the diagnostic groups; (iv) to perform an enriched pathway analysis of the significantly dysregulated miRNAs; and finally (v) to apply the PBM approach using Parkinson’s disease map to understand the complex dynamics of disease models while simulating the downstream signalling effects of dysregulated miRNAs.

## Materials and methods

### Recruitment and ethical considerations

All participants enrolled into the Luxembourg Parkinson's Study agreed and signed a written informed consent. The study has been approved by the National Research Ethics Committee (CNER Ref: 201407/13) and complied with the Declaration of Helsinki. The Luxembourg Parkinson's Study was registered in ClinicalTrials.gov under NCT05266872.

### Group definition and selection of study individuals

**Figure 1:**
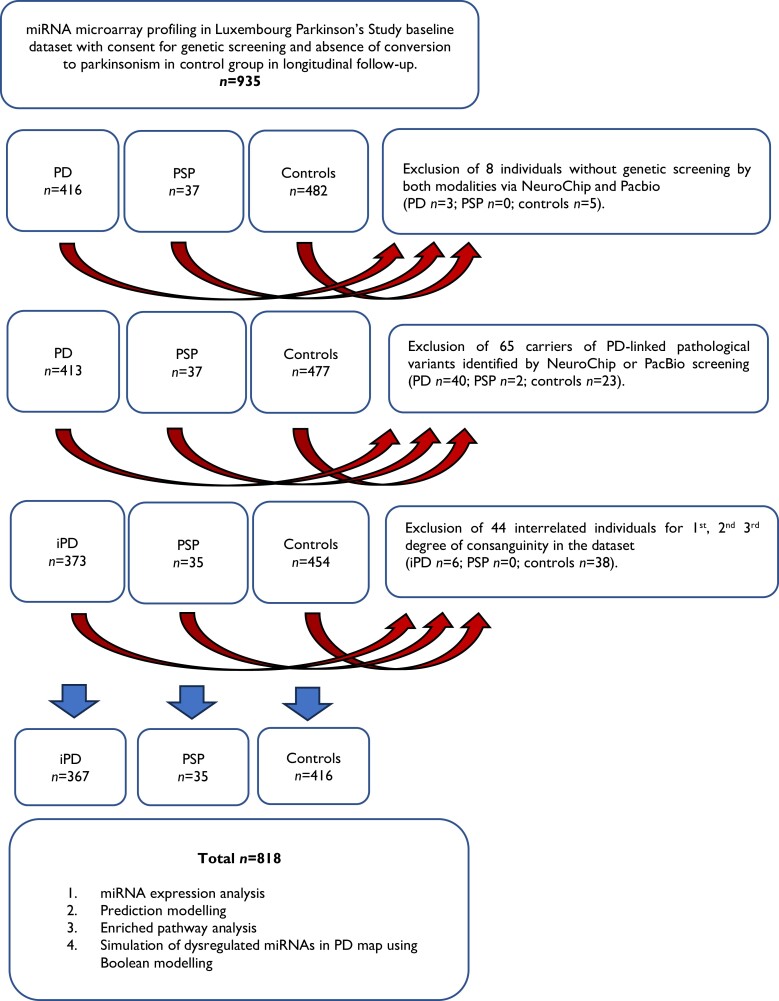
**Flowchart describing selection criteria and analytical pipeline in Luxembourg Parkinson's Study.** PD, Parkinson's disease; iPD, idiopathic Parkinson’s disease; PSP, progressive supranuclear palsy; miRNA, microRNA.

The design, recruitment and baseline characteristics of the Luxembourg Parkinson's Study were previously published in detail.^33,38^ Diagnosis of Parkinson’s disease was based on the diagnostic criteria UK Parkinson's Disease Society Brain Bank Diagnostic Criteria (UKPDSBB) without consideration of more than one positive family history of Parkinson’s disease as an exclusion criterion.^39^ For progressive supranuclear palsy, we used the Movement Disorder Society criteria from 2017.^40^ The group of controls was defined as individuals older than 18 years without evidence for an active cancer, pregnancy or clinical evidence (and imaging, where available) for a neurodegenerative disorder. Data export from electronic database REDCap (baseline visit) was performed on 10 June 2023. In total, 818 individuals with miRNA microarray data were included into the analysis after exclusion due to (i) the lack of genetic screening modalities by either NeuroChip (Illumina) or PacBio^37^; (ii) the presence of Parkinson’s disease–linked mutations; or (iii) the presence of blood-interrelated individuals in the data set as described in detail in Fig. 1.

### Clinical assessment and data

Sociodemographic and clinical characteristics for Parkinson’s disease, progressive supranuclear palsy and controls were chosen from the basic clinical assessment battery and are listed in Table 1. All patients were evaluated in ON state. Family history of parkinsonism or dementia and comorbidities were determined during a semi-structured interview during the assessment with a study physician.

**Table 1:** Descriptive statistics of sociodemographic and clinical profile of investigated groups with available miRNA microarray data in Luxembourg Parkinson's Study

	Control *n* = 416			Parkinson’s disease *n* = 367			Progressive supranuclear palsy *n* = 35			*P*-value^a^		
Clinical/demographic variables	Mean or yes in %	SD or no/yes	NA	Mean or yes in %	SD or no/yes	NA	Mean or yes in %	SD or no/yes	NA	Controls versus Parkinson’s disease	Controls versus progressive supranuclear palsy	Parkinson’s disease versus progressive supranuclear palsy
Sex (male)^b^	56%	182/234	0	68%	116/251	0	57%	15/20	0	5.2*e*−04*	1.0*e*+00	1.9*e*−01
AAO (years)			416	61.96	11.91	0	68.51	7.22	0			5.3*e*−04*
AAA (years)	58.39	11.63	0	67.54	11.18	0	71.51	6.17	0	1.3*e*−25*	1.6*e*−11*	3.0*e*−02
Disease duration since diagnosis (years)			416	5.62	5.49	0	3.00	2.49	0			1.0*e*−02
Years of education	14.26	3.76	3	12.86	4.10	0	10.77	3.68	0	4.3*e*−07*	1.1*e*−06*	6.7*e*−03
Total languages spoken	3.50	0.81	0	2.89	1.05	0	2.80	0.83	0	3.0*e*−19*	4.8*e*−08*	3.4*e*−01
Family history of parkinsonism^b^	27%	302/112	2	25%	277/90	0	18%	28/6	1	4.6*e*−01	3.1*e*−01	5.3*e*−01
Family history of dementia^b^	31%	285/126	5	22%	284/82	1	24%	26/8	1	1.2*e*−02	4.4*e*−01	8.3*e*−01
Hoehn and Yahr scale	0	0.00	2	2.27	0.86	2	3.11	1.37	0	4.7*e*–153*	3.2*e*−99*	3.5*e*−04*
MDS–UPDRS III	3.23	4.75	6	35.73	17.81	9	49.60	22.11	0	4.1*e*−121*	2.5*e*−23*	5.5*e*−04*
LEDD (g/day)	0.0046	0.043	0	0.55	0.42	0	0.55	0.41	1	6.7e-126*	2.4*e*−73*	9.1*e*−01
MoCA	27.10	2.44	2	24.03	4.64	9	19.55	7.61	2	1.8*e*−25*	3.2*e*−13*	2.8*e*−04*
Diabetes^b,c^	6%	393/23	0	11%	328/39	0	20%	28/7	0	1.1*e*−02	5.1*e*−03	1.0*e*−01
Arterial hypertension^b^	31%	287/129	0	44%	207/160	0	54%	16/19	0	2.8*e*−04*	7.8*e*−03	2.9*e*−01
Cardiovascular disease^b^	10%	375/41	0	22%	285/82	0	14%	30/5	0	1.9*e*−06*	3.8*e*−01	3.9*e*−01
Hypercholesterolaemia^b^	35%	272/144	0	42%	214/153	0	31%	24/11	0	4.6*e*−02	8.5*e*−01	2.8*e*−01
History of stroke^b^	2%	406/10	0	5%	348/19	0	17%	29/6	0	5.6*e*−02	6.5*e*−04*	1.5*e*−02

NA, number of missing values; SD, standard deviation; MDS–UPDRS III, Movement Disorder Society–Unified Parkinson's Disease Rating Scale, part III; LEDD, levodopa equivalent daily dose; MoCA, Montreal Cognitive Assessment.

^a^ *P*-values from Mann–Whitney U-test for numerical variables and Fisher's exact test for binary variables.

^b^Binary variables.

^c^Type of diabetes not defined.

^*^Statistically significant at Bonferroni-adjusted 5% level.

### Missing data statement

The absolute number and percentage of missing values per variable and per group is listed in Table 1. Due to a low proportion of missing data, we used a pairwise deletion for all statistical models.

### miRNA analysis and quality control

The process of miRNA identification via microarray was previously described in detail.^41^ In short, RNA was extracted from all whole blood samples using the PAXgene miRNA Kit (Qiagen) according to the manufacturer's instructions. RNA quality and quantity were evaluated using a Bioanalyzer 2100 Instrument (Agilent Technologies) and NanoDrop ND-1000, respectively. Microarray screening of high-quality RNA samples was performed on Agilent's SureScan DX Microarray Scanner following the manufacturer's instructions and as described previously.^42-44^ Each array targeted 2549 miRNAs with 20 replicates per probe with further detailed procedure description published previously.^26,45^

### Genotyping and quality control analyses

The methods for genotyping in our data set have been described previously.^46^ Parkinson’s disease–causing rare variants were defined by the ClinVar classification as ‘pathogenic/likely pathogenic’. The ethnic origin of all individuals within the Luxembourg Parkinson's Study included in this study clustered strongly with European ancestry as comprehensively analysed in our previous work.^47^

### Statistical analysis

Descriptive statistics, differential expression analysis and predictive modelling were performed using R statistical programming language version 4.3.0. Descriptive statistics of the investigated diagnostic groups (idiopathic Parkinson’s disease, progressive supranuclear palsy and controls) was represented in mean and standard deviation (SD) for numerical variables and number of NO/YES for binary ones. Intergroup comparisons were performed using Mann–Whitney U-test and Fisher's exact test for numerical variables and binary variables, respectively (Table 1). Moderated *t*-test implemented in the R package limma was used to test the effects of idiopathic Parkinson’s disease versus controls, progressive supranuclear palsy versus controls and progressive supranuclear palsy versus idiopathic Parkinson’s disease on miRNA expression, adjusted for age and sex using multiple linear regression (MLR). For each diagnostic group, we also examined the effect of age on miRNA expression, adjusted for sex, and the effect of sex on miRNA expression, adjusted for age. At all instances, false discovery rate (FDR; Benjamini–Hochberg) and family-wise error rate (FWER; Bonferroni) correction for multiple testing were applied. We considered a miRNA to be significantly dysregulated at FDR, and sociodemographic characteristics or clinical outcomes were considered significant at FWER. Jaccard index was calculated for all comparisons to investigate an intersection between nominally significant (*P* ≤ 0.05) miRNAs. Finally, cross-validated area under the receiver operating characteristic (ROC) curve (AUC) was calculated from logistic lasso regression (for binary outcomes) for a maximum number of miRNAs from 0 to 100 (AUC shown for 0, 10 and 100 miRNAs) with and without inclusion of age and sex in the prediction model. Early-stage idiopathic Parkinson’s disease and earlier-stage progressive supranuclear palsy were defined by disease duration since diagnosis ≤ 5 years.

The statistical pathway analyses of the miRNA data for idiopathic Parkinson’s disease, progressive supranuclear palsy and controls were performed using the R statistical programming language (version 4.2.0, RRID:SCR_001905) and the PanomiR package for miRNA mapping and pathway activity profile generation (version 1.1.2).^48^ First, Entrez/pathway mappings were retrieved from the MSigDB database (C2 collection of curated pathway gene sets),^34^ and mappings between miRNAs and their target genes in Entrez format were obtained from the TargetScan database (RRID:SCR_010845).^49^ Next, the FDR-adjusted significant miRNAs with *P* ≤ 0.05 for each pairwise comparison between conditions (idiopathic Parkinson’s disease versus controls, progressive supranuclear palsy versus idiopathic Parkinson’s disease and progressive supranuclear palsy versus controls) were used to determine their target genes. Following this, an enrichment analysis was conducted for the target genes of each miRNA in the MSigDB pathways using the function ‘miRNAPathwayEnrichment’ in the PanomiR package and setting the minimum pathway size to 10. The results for the miRNA/pathway combinations were ranked by increasing *P*-value, and adjusted *P*-values were determined using FDR.^50^

PBM was applied to represent the complex molecular mechanisms underlying idiopathic Parkinson’s disease and progressive supranuclear palsy. PBM was fitted on the Parkinson’s disease map, a comprehensive molecular interaction diagram that captures the key mechanisms involved in the initiation and progression of Parkinson’s disease.^35^ The Parkinson’s disease map was translated into PBM in an automated fashion using the significantly dysregulated miRNAs (at FDR) identified in MLR of idiopathic Parkinson’s disease versus controls and progressive supranuclear palsy versus controls. This translation was essential for simulating the downstream signalling effects of dysregulated target molecules in idiopathic Parkinson’s disease and progressive supranuclear palsy when compared to controls. By abstracting the disease mechanisms into a logical form by using PBM, it allowed simulations of disease dynamics. PBM approach used a series of random walks to determine the probability of components within the model.^51^ The PBM combined the qualities of both discrete and continuous Markov processes, within a Monte Carlo framework.^52^ To establish a foundational baseline for our simulations, initial state probabilities were parameterized in the PBM. This step was critical as it sets the starting point for the model, reflecting the pre-simulation status of molecular interactions within the Parkinson’s disease framework. The equation for updating the state probabilities is given by:


$$Pt+1(s)=\sum s^{\prime }\in SPt(s^{\prime })\cdot P(s^{\prime }|s)$$

where Pt+1(s) is the probability of state *s* at the next time point and $P(s^{\prime }|s)\;$ is the transition probability from a previous state $s^{\prime }$ to the current state *s*. The goal was to accurately predict the temporal fluctuations in the model. In addition, we used the multiple change point regression algorithm to detect inflection points throughout the iterative steps. This approach allowed to identify the significant alterations within the model's behaviour during the simulation.^36^ The sensitivity analysis was performed to evaluate the impact of molecular perturbations in Boolean models, focusing on how perturbations affect the model's dynamics and stability as described in details in the Supplementary material.

## Results

### Study population

**Figure 2:**
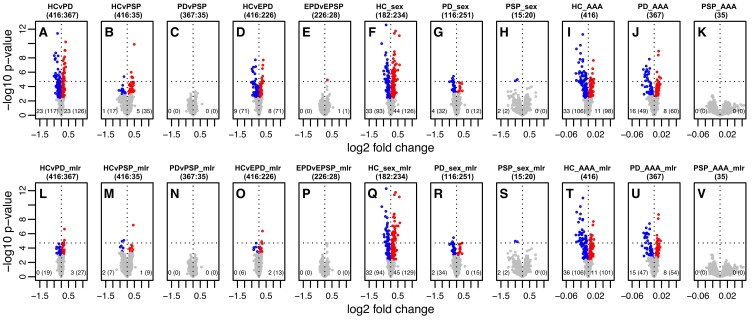
**miRNA expression analysis in Luxembourg Parkinson's Study.** Volcano plots showing minus log_10_ transformed *P*-values (significance, *y*-axis) against log_2_ fold change (effect size, *x*-axis) of miRNA expression assessed by microarray. The first row shows miRNA expression without adjustment for confounders (**A-K**). The second row shows results of MLR adjusting for confounders (**L to P** adjusted for age and sex; **Q to S** adjusted for age; **T to V** adjusted for sex). Sample size per group and per comparison is shown in brackets next to the plot designation letter. Significant *P*-values at FWER of 5% (Bonferroni) are above the dashed line, and significant *P*-values at an FDR of 5% (Benjamini–Hochberg) are highlighted in colour. Significantly upregulated and downregulated are highlighted in red and blue colours, respectively. Number of significantly dysregulated miRNA(s) in bottom of each plot at FWER and at FDR in brackets (left corner, negative effect size; right corner, positive effect size). iPD, idiopathic Parkinson's disease; EPD, early-stage Parkinson’s disease; EPSP, earlier-stage progressive supranuclear palsy; HC, controls; PSP, progressive supranuclear palsy; AAA, age at assessment; sex, female/male.

A total of 818 individuals from the Luxembourg Parkinson's Study were included in the study (idiopathic Parkinson’s disease *n* = 367; progressive supranuclear palsy *n* = 35; and controls *n* = 416) after applying all exclusion criteria visualized in Fig. 1. The results of the miRNA expression analysis are shown in Fig. 2, and all significantly dysregulated miRNAs per model with *P*-value and effect size are listed in Tables 2 and 3 and Supplementary Tables 1–5 and 7–13. Intergroup comparison revealed idiopathic Parkinson’s disease and progressive supranuclear palsy to be significantly older than controls (mean age ± SD, 67.54 ± 11.18 and 71.51 ± 6.17 versus 58.39 ± 11.63 years, both *P* < 0.001) with significantly higher proportion of males in idiopathic Parkinson’s disease versus controls (68% versus 56%, *P* < 0.001). Age at onset (AAO) was significantly higher in progressive supranuclear palsy versus idiopathic Parkinson’s disease (68.51 ± 7.22 versus 61.96 ± 11.9 years, *P* < 0.001). With regard to the comorbidities, reported arterial hypertension and cardiovascular disease were significantly more frequent in idiopathic Parkinson’s disease versus controls (44% versus 31%, *P* < 0.001, and 22% versus 10%, *P* < 0.001, respectively), and progressive supranuclear palsy versus controls had higher frequency of stroke (5% versus 2%, *P* < 0.001). Remaining sociodemographic characteristics, comorbidities and quantitative markers of disease stage and severity are presented in Table 1.

**Table 2:** Significantly dysregulated miRNAs identified in multiple linear regression analysis of Parkinson's disease versus controls

miRNA upregulated *n* = 27; downregulated *n* = 19	Effect size	*P*-value
miR-145-5p	0.219	2.3*e*−07**
miR-151a-3p	0.218	8.0*e*−06**
miR-3677-5p	0.165	1.8*e*−05**
miR-145-3p	0.069	2.2*e*−05*
miR-130a-3p	−0.170	2.5*e*−05*
miR-491-5p	0.108	2.8*e*−05*
miR-4516	0.177	2.9*e*−05*
**miR-197-3p^a^**	0.180	3.9*e*−05*
**miR-505-3p^a^**	0.160	4.2*e*−05*
miR-1285-5p	0.090	6.4*e*−05*
miR-4538	−0.104	6.6*e*−05*
miR-18b-5p	−0.325	7.2*e*−05*
miR-539-3p	0.066	7.8*e*−05*
miR-122-5p	−0.163	8.7*e*−05*
miR-18a-5p	−0.338	1.2*e*−04*
miR-103a-3p	−0.116	1.7*e*−04*
miR-326	0.203	1.7*e*−04*
miR-3923	−0.084	1.8*e*−04*
miR-3155a	0.071	1.8*e*−04*
**miR-1225-5p^a^**	0.091	2.0*e*−04*
miR-937-5p	−0.096	2.4*e*−04*
miR-3145-5p	0.068	2.6*e*−04*
miR-125a-5p	0.188	2.7*e*−04*
miR-6720-5p	0.133	2.8*e*−04*
miR-451a	−0.018	3.0*e*−04*
**let-7d-3p^a^**	0.150	3.1*e*−04*
miR-7106-5p	−0.166	3.2*e*−04*
miR-328-3p	0.168	3.5*e*−04*
miR-6799-5p	−0.144	4.1*e*−04*
miR-23a-3p	0.150	4.5*e*−04*
miR-6770-3p	0.099	4.6*e*−04*
miR-6849-5p	−0.137	4.6*e*−04*
miR-4323	0.127	4.7*e*−04*
miR-762	−0.097	5.1*e*−04*
miR-590-5p	−0.327	5.2*e*−04*
miR-138-2-3p	−0.110	5.2*e*−04*
miR-6716-5p	0.150	5.4*e*−04*
miR-922	0.188	5.4*e*−04*
miR-4328	0.056	5.7*e*−04*
miR-20b-5p	−0.233	6.2*e*−04*
miR-423-3p	0.129	6.6*e*−04*
miR-4281	0.103	6.7*e*−04*
miR-378a-5p	0.175	7.0*e*−04*
miR-6727-5p	−0.119	8.0*e*−04*
miR-4513	−0.175	8.4*e*−04*
miR-662	−0.066	8.9*e*−04*

Log fold changes and *P*-values for effect in Parkinson's disease versus controls (model HCvPD_mlr; *n* = 367 versus 416). No effect implies fold change = 1 and log fold change = 0. Significantly upregulated and downregulated miRNAs are demonstrated by positive and negative effect sizes, respectively.

^a^Overlapping miRNAs in significance and direction between Parkinson’s disease versus controls and progressive supranuclear palsy versus controls are highlighted in bold.

^*^Significant at an FDR of 5% (Benjamini–Hochberg).

^**^Significant at an FWER of 5% (Bonferroni) across all comparisons.

**Table 3:** Significantly dysregulated miRNAs identified in multiple linear regression analysis of progressive supranuclear palsy versus controls

miRNA upregulated *n* = 9; downregulated *n* = 7	Effect size	*P*-value
miR-2115-5p	0.424	6.4*e*−08**
miR-4762-3p	−0.228	8.3*e*−06**
miR-7975	−0.357	1.2*e*−05*
miR-4270	0.298	3.7*e*−05*
miR-1233-5p	−0.413	5.2*e*−05*
**miR-505-3p^a^**	0.353	5.5*e*−05*
miR-6085	−0.486	7.4*e*−05*
miR-125a-3p	−0.283	9.8*e*−05*
miR-769-5p	0.289	1.0*e*−04*
miR-3065-3p	0.164	1.2*e*−04*
miR-4638-5p	0.169	1.4*e*−04*
**miR-197-3p^a^**	0.391	1.6*e*−04*
miR-4465	−0.362	2.6*e*−04*
**let-7d-3p^a^**	0.328	3.0*e*−04*
miR-564	−0.339	3.1*e*−04*
**miR-1225-5p^a^**	0.208	3.1*e*−04*

Log fold changes and *P*-values for effect in progressive supranuclear palsy versus controls (HCvPSP_mlr; *n* = 35 versus 416). No effect implies fold change = 1 and log fold change = 0. Significantly upregulated and downregulated miRNAs are demonstrated by positive and negative effect sizes, respectively.

^a^Overlapping miRNAs in significance and direction between Parkinson’s disease versus controls and progressive supranuclear palsy versus controls are highlighted in bold.

^*^Significant at an FDR of 5% (Benjamini–Hochberg).

^**^Significant at an FWER of 5% (Bonferroni) across all comparisons.

### Age and sex-related miRNA expression analysis

Given the significant differences in age and sex between the groups, we first performed a differential miRNA expression analysis of age and sex in each diagnostic group and intersection of potential overlap between age- and sex-related significantly dysregulated miRNAs. As detailed in Fig. 2F to K and Q to V, sex and age showed a significant dysregulation of multiple miRNAs. When investigating the effect of sex on miRNA expression adjusted for age, 223 miRNAs were found significantly dysregulated in controls (129 upregulated and 94 downregulated, listed in Supplementary Table 1), 49 miRNAs in idiopathic Parkinson’s disease (15 upregulated and 34 downregulated, listed in Supplementary Table 2), and 2 miRNAs in progressive supranuclear palsy group (both downregulated, listed in Supplementary Table 3).

Regarding the effect of age on miRNA expression adjusted for sex, 207 miRNAs were found to be significantly dysregulated in controls (101 upregulated and 106 downregulated, listed in Supplementary Table 4), 101 miRNAs in idiopathic Parkinson’s disease (54 upregulated and 47 downregulated, listed in Supplementary Table 5) and none in progressive supranuclear palsy group. Jaccard index for overlapping age- and sex-associated nominally significant miRNA did not indicate a high overlap (idiopathic Parkinson’s disease 8%, controls 19%, progressive supranuclear palsy 2%; see Supplementary Table 6), indicating that most of the significantly dysregulated miRNAs by age are not associated with sex and vice versa.

### miRNA expression analysis and prediction model of idiopathic Parkinson’s disease versus controls

**Figure 3:**
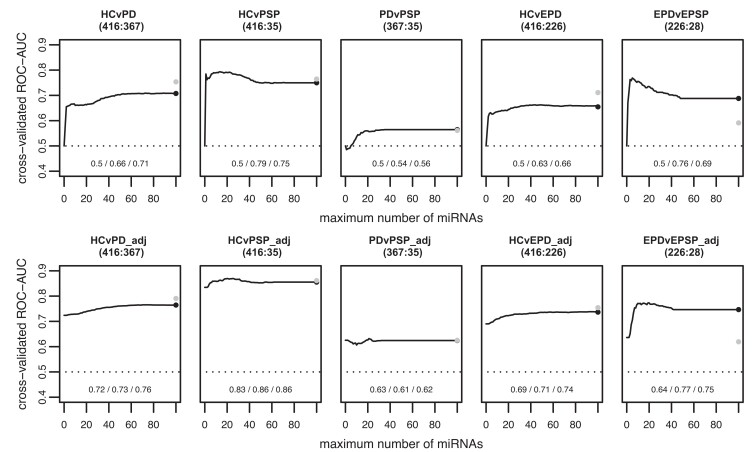
**Predictive modelling between disease groups and controls using miRNA subsets.** Cross-validated AUC from logistic lasso regression (binary outcomes) on the *y*-axis against maximum number of miRNAs on the *x*-axis for intergroup prediction of idiopathic Parkinson's disease (*n* = 367), HC (*n* = 416) and progressive supranuclear palsy (*n* = 35). Early-stage Parkinson’s disease (*n* = 226) and earlier-stage progressive supranuclear palsy (*n* = 28) were defined by disease duration since diagnosis ≤ 5 years. The second row also includes the unpenalized covariates age and sex in the models (HCvPD_adj, HCvPSP_adj and PDvPSP_adj). The numbers at the bottom indicate the AUC with at most 0, 10 and 100 miRNAs (non-zero coefficients other than intercept, AAA and sex). The black and grey dots show the predictive performance of standard lasso and ridge regression, respectively.

Fitting MLR to examine the effect of idiopathic Parkinson’s disease versus controls on miRNA expression adjusted for age and sex revealed 46 significantly dysregulated miRNAs (27 upregulated and 19 downregulated) as shown in Fig. 2 (model ‘HCvPD_mlr’), and significant miRNAs are listed in Table 2. When using miRNAs to predict the diagnostic status between idiopathic Parkinson’s disease and controls, we achieved AUC of 0.5/0.66/0.71 including at most 0/10/100 miRNAs and AUC of 0.72/0.73/0.76 when using at most 0/10/100 miRNAs and unpenalized variables age and sex in the prediction model (Fig. 3, models ‘HCvPD’ and ‘HCvPD_adj’, respectively).

### miRNA expression analysis and prediction model of progressive supranuclear palsy versus controls

In miRNA expression analysis of progressive supranuclear palsy versus controls using MLR adjusted for age and sex, 16 miRNAs were found to be significantly dysregulated (9 upregulated and 7 downregulated; see Fig. 2, model ‘HCvPSP_mlr’). All significant miRNAs are listed in Table 3. Out of these, four miRNAs overlapped in significance and direction when compared to idiopathic Parkinson’s disease versus controls (*miR-197-3p*, *miR-505-3p*, *miR-1225-5p* and *let-7d-3p*, all upregulated in both comparisons). The logistic lasso regression in progressive supranuclear palsy versus controls with at most 0/10/100 miRNAs was 0.5/0.79/0.75 and AUC of 0.83/0.86/0.86 with at most 0/10/100 miRNAs and unpenalized age/sex in the model (Fig. 3, models ‘HCvPSP’ and ‘HCvPSP_adj’, respectively).

### miRNA expression analysis and prediction model of idiopathic Parkinson’s disease versus progressive supranuclear palsy

No significantly dysregulated miRNAs were identified in MLR of idiopathic Parkinson’s disease versus progressive supranuclear palsy with adjustment for age and sex (Fig. 2, ‘PDvPSP_mlr’). As expected, predictive power of at most 0/10/100 miRNAs was very low with AUC 0.5/0.54/0.56 and AUC 0.63/0.61/0.62 with at most 0/10/100 miRNAs and unpenalized age/sex in the model (Fig. 3, models ‘PDvPSP’ and ‘PDvPSP_adj’, respectively). However, when comparing earlier-stage progressive supranuclear palsy versus early-stage idiopathic Parkinson’s disease in the model using at most 0/10/100 miRNAs and unpenalized age/sex, we achieved AUC 0.64/0.77/0.75, hence comparable to the AUC in predicting idiopathic Parkinson’s disease from controls (see Fig. 3).

### Targeted sub-analysis of miRNA subset

Given the substantial variation in the reported significantly dysregulated miRNAs across published studies, we performed a sub-analysis of all 13 miRNAs with a cross-study significance identified in a large meta-analysis of Parkinson’s disease versus controls.^27^ Out of the 13 miRNAs, we replicated 3 miRNAs, 2 in terms of significance and direction (*miR-141-3p* and *miR-451a*, both downregulated) and 1 with various directions reported across the studies (*miR-185-5p*, upregulated in our study). While the reference study did not adjust the models for age and sex, we inquired in our study whether the remaining 10 dysregulated miRNAs were significantly dysregulated solely due to the covariates age or sex.

Focusing on the models adjusting for age or sex in Parkinson’s disease and controls (see Supplementary Table 14, models with MRL), five miRNAs were associated with age and/or sex in Parkinson’s disease, controls or both as follows: (i) *miR-221-3p* was significantly negatively associated with age in Parkinson’s disease and in controls; (ii) *miR-29a-3p* was significantly positively associated with age in Parkinson’s disease and significantly negatively associated with male sex in controls; (iii) *miR-193a-3p* was significantly positively associated with male sex in controls; (iv) *miR-133b* was significantly negatively associated with male sex in Parkinson’s disease and controls; and (v) m*iR-15b-5p* was significantly negatively associated with age in Parkinson’s disease and controls. Remaining 5 out of 10 miRNAs were not significantly associated with age/sex in Parkinson’s disease or in controls (*miR-214-3p*, *miR-29c-3p*, *miR-19b-3p*, *miR-146a-5p* and *miR-181a-5p*), and we did not validate the previous findings as reported in the reference study.^27^

### Enriched pathway analysis of dysregulated miRNAs

In order to inquire into the molecular pathways influenced by the significantly dysregulated miRNAs, we performed an enriched pathway analysis mapping miRNAs and their target genes using MSigDB. Though none of the pathway remained significant after the correction for multiple comparisons, the highest ranked dysregulated pathway was ‘BioCarta natural killer (NK) cell pathway’ (idiopathic Parkinson’s disease versus controls, *P*-value 0.0000487, adjusted *P*-value 0.527; progressive supranuclear palsy versus controls, *P*-value 0.0000487, adjusted *P*-value 0.105; see Supplementary material referring to enriched pathway analysis).

### Pathway analysis of dysregulated miRNAs using PBM in Parkinson’s disease map

Considering the significantly dysregulated miRNAs (significant at FDR) identified in the MLR models of idiopathic Parkinson’s disease versus controls and progressive supranuclear palsy versus controls, we performed a PBM using the Parkinson’s disease map. In comparison to the enriched pathway analysis reported above, this system biology approach allowed us to gain a mechanistic understanding of the dysfunction in the cellular processes based on the most-recent knowledge of pathophysiology in Parkinson’s disease. We simulated the effects of dysregulated miRNAs in the Parkinson’s disease map by identifying their target molecules and estimating their impact on relevant pathways, as detailed in Tables 4 and 5. Interestingly, we identified the following overlapping pathways affected by miRNAs in Parkinson’s disease versus controls and progressive supranuclear palsy versus controls: activity of transcription factor EB (TFEB); endoplasmic reticulum (ER) stress signalling; calcium signalling pathway; dopaminergic transcription; and peroxisome proliferator-activated receptor gamma coactivator-1α (PPARGC1) activity. Though the simulated intermediate molecules differed substantially between idiopathic Parkinson’s disease and progressive supranuclear palsy, the simulated impact on the cellular dysfunction predicted a partially convergent dysfunction of the lysosomal activity and autophagy, ER-associated degradation (ERAD), calcium homeostasis, mitochondrial respiration and mitochondrial biogenesis. The complete list of dysregulated pathways in idiopathic Parkinson’s disease versus controls (i.e. including the non-overlapping ones with progressive supranuclear palsy versus controls), target molecules and their simulated cellular behaviour is included in Supplementary Table 15.

**Table 4:** Target simulation for significantly dysregulated miRNAs in Parkinson's disease versus controls using Boolean modelling applied in the Parkinson’s disease map

Pathway category	Dysregulated molecules	Simulated elements	Simulated behaviour	Interpretation
TFEB activity	MITF, PTEN, WDR45, RAB7A, PRKAG2, BECN1, ATP6VOD1, ATP6V1H, ATP6V1E1, ATP6V0E1, ATP6V1C1	Lysosomal acidification; autophagy	Impaired acidification; compromised autophagy	Alterations in TFEB activity affect autophagy and lysosomal function, crucial for the degradation of α-Syn aggregates in Parkinson’s disease.
ER stress signalling	CEBPB, DDIT4, MAP1LC3B, BECN1, HSP90B1	Autophagy; unfolded protein response (UPR); chaperone activity	Impaired autophagy; activated UPR; modified chaperone activity	Chronic ER stress and the unfolded protein response contribute to the pathogenesis of Parkinson’s disease by affecting protein folding and clearance mechanisms
Calcium signalling	ATP1A1, ATP1B1, SLC8A2, CACNA1D, CACNA2D2, CACNA2D3, CACNB2, ERN2, EIF2AK2, CAMK2, CAMK2B, PPP3CA, PPP3CB, YWHAB, RPS6KA1, RPS6KA3	Calcium homeostasis; CREB signalling	Disrupted calcium homeostasis; altered CREB signalling	Dysregulation of calcium signalling can lead to neuronal excitotoxicity and affect various signalling pathways implicated in Parkinson’s disease
Dopaminergic transcription	NR4A2, NCOR2, FOXA2, SNCA, PARK7(DJ1), CGH1, TH, DDC, SLC18A, ALC6A3, CFLAR, SOD1, COX5B, COX6A1, NDUFB8, BDNF, RET, ALDH1A1, FOXO1, FOXO3, KLC1, MAP1B, EN1, FOXA1, FOXA2	Transcriptional regulation; mitochondrial function	Dysregulated transcription; compromised mitochondrial function	Impairment in the transcriptional machinery and mitochondrial dysfunction are key features in the loss of dopaminergic neurons in Parkinson’s disease.
PPARGC1A activity	SIRT1, ESR, IDH3B, IDH3G, COX5B, COX6A, COX6B1, COX7A2, COX7C, SDHB, TOMM20, SURF1, VDAC1, TFB2M	Mitochondrial biogenesis; respiratory function	Altered mitochondrial biogenesis; impaired respiratory function	PPARGC1A is a key regulator of mitochondrial biogenesis and function, and its dysregulation is implicated in the mitochondrial dysfunction observed in Parkinson’s disease.

The pathway categories (*n* = 5) overlapping between Parkinson’s disease versus controls and progressive supranuclear palsy versus control are shown. Column ‘dysregulated molecules’ refers to the target molecules within the dysregulated pathway(s) in the simulation. Column ‘simulated elements’ reflects on the broader impact at the cellular or system level highlighting the elements that are simulated or affected due to the molecular dysregulation. Column ‘simulated behaviour’ describes the consequent aberrant behaviour or states induced by the pathway dysregulation(s).

**Table 5:** Target simulation for significantly dysregulated miRNAs in progressive supranuclear palsy versus controls using Boolean modelling applied in the Parkinson's disease map

Pathway category	Dysregulated molecules	Simulated elements	Simulated behaviour	Interpretation
TFEB activity	ARP6V0B, CLN3, GLA	Lysosomal acidification; lysosomal membrane organization	Reduced acidification; disrupted membrane organization	Disruption in TFEB-mediated autophagy and lysosomal biogenesis impairs cellular waste management, contributing to mitochondrial dysfunction
ER stress signalling	SIGMAR1, ATG7, EIF2AK3	Autophagy; ERAD; SIGMAR1	Reduced autophagy; enhanced ERAD; stable SIGMAR1 activity; EIF2AK3	ER stress alters cellular survival and apoptotic pathways. Dysregulation in autophagy and ERAD may contribute to neuronal death in progressive supranuclear palsy
Calcium signalling	CAMK2G, RPS6KA3, NCS1	CREB1; TORC1 and TORC2; NR4A2; PPARGC1; dopamine receptor endocytic activity	Inhibited CREB1; inhibited TORC1/2; downregulated NR4A2; downregulated PPARGC; reduced dopamine receptor endocytic activity	Calcium signalling dysregulation disrupts neuronal transcriptional responses, exacerbating progressive supranuclear palsy's neurodegenerative process
Dopaminergic transcription	COX8A	Mitochondrial biogenesis	Dysregulated biogenesis	COX8A dysfunction underpins compromised mitochondrial health, critical for maintaining dopaminergic neuron integrity in progressive supranuclear palsy
PPARGC1 activity	SURF1, COX8A	Mitochondrial transport	Accelerated mitochondrial transport	SURF1 overexpression, likely compensatory, affects mitochondrial dynamics, essential for preserving neuronal health amidst progressive supranuclear palsy pathology

Pathway categories (*n* = 5) overlapping between Parkinson’s disease versus controls and progressive supranuclear palsy versus control are shown. Column ‘dysregulated molecules’ refers to the target molecules within the dysregulated pathway(s) in the simulation. Column ‘simulated elements’ reflects on the broader impact at the cellular or system level highlighting the elements that were simulated or affected due to the molecular dysregulation. Column ‘simulated behaviour’ describes the simulation of the consequent aberrant behaviour or states induced by the pathway dysregulation(s).

## Discussion

### Replication of miRNAs across the studies

This study represents a comprehensive analysis of 2549 miRNA expression profiles and target pathway analysis of dysregulated miRNAs in a large data set of individuals with idiopathic Parkinson’s disease, controls and prototypic tauopathy progressive supranuclear palsy from the Luxembourg Parkinson's Study. Given the accumulating number of reports on miRNA profiles in Parkinson’s disease lacking a replication of the findings across multiple independent data sets, we compared our results with a large meta-analysis of blood-derived miRNA profiles. In the reference meta-analysis,^27^ 13 miRNAs were found with study-wide significance across at least 3 independent data sets (10 significant in the same direction and 3 significant with inconsistent direction across the meta-analysed studies) when comparing Parkinson’s disease with controls. Out of these 13 dysregulated miRNAs, we replicated the results for 3 miRNAs in our study (*miR-141-3p* and *miR-451a* dysregulated in the same direction; *miR-185-5p* with inconsistently reported direction; see Supplementary Table 14, model ‘HCvPD’). Interestingly, one study demonstrated *miR-141-3p* to be implicated in the mitochondrial dysfunction, apoptosis and oxidative stress in 1-methyl-4-phenylpyridinium–treated induced cell model of Parkinson’s disease.^53^ Though it aligns well with increasing body of evidence highlighting the substantial role of mitochondrial dysfunction in the pathogenesis of Parkinson’s disease, this cellular model did not account for the chronic progressive neurodegeneration observed in Parkinson’s disease but rather modelled an acute damage of dopaminergic neurons.^7^ Additionally, the second replicated *miR-451a* was recently found to be dysregulated in the prodromal stage of Parkinson’s disease in idiopathic rapid eye movement (REM) sleep behaviour disorder (iRBD)^54^ as well as consistently dysregulated in non-manifesting *LRRK2* mutation carriers and idiopathic Parkinson’s disease individuals.^55^ Additionally, the second replicated *miR-451a* was recently found to be dysregulated in the prodromal stage of Parkinson’s disease in iRBD as well as consistently dysregulated in non-manifesting *LRRK2* mutation carriers and idiopathic Parkinson’s disease individuals. As it was shown that *miR-451a* levels are highly susceptible to haemolysis of the blood sample(s),^56^ the fact that upregulated *miR-451a* was found and replicated across multiple independent studies and replicated in above-discussed large meta-analysis in Parkinson’s disease versus controls or in at-risk cohorts versus controls (iRBD or mutation carriers), argues against a systematic bias due to haemolysis in our study. As for the remaining study-wide significant miRNAs reported in the referenced meta-analysis, we inquired into the possible significant dysregulation of the remaining 10 miRNAs due to the age/sex itself, rather than the disease, since the meta-analysis did not adjust the models for these confounders. Indeed (as shown in Table 4), 5 out of all 13 study-wide significant miRNAs were significantly associated with age and/or sex in idiopathic Parkinson’s disease and/or controls. This observation underpins the important role of carefully adjusting for the effect of age and sex in the miRNA expression analysis and should be well accounted for in all future studies.

### Partial functional convergence between idiopathic Parkinson’s disease and progressive supranuclear palsy

It has been suggested that pathologically distinct classes of neurodegenerative disorders might converge at the molecular level in terms of pathophysiology, i.e. in terms of cause(s) or consequence(s) of the neurodegeneration.^57^ In support of the functional convergence, we identified four significantly dysregulated miRNAs that overlap in direction between idiopathic Parkinson’s disease versus controls and progressive supranuclear palsy versus controls (*miR-197-3p*, *let-7d-3p*, *miR-1225-5p* and *miR-505-3p*, all four upregulated). From the four overlapping dysregulated miRNAs in our data set, we validated the upregulated *let-7d-3p* when compared to a large meta-analysis of miRNAs across the studies for Alzheimer’s disease focusing on the dysregulated miRNAs with study-wide significance across meta-analysed studies.^58^ Additionally, significantly upregulated *miR-505-3p* in our data set was previously identified in plasma of Parkinson’s disease versus controls (detection method: TaqMan microarray)^59^ and Alzheimer’s disease versus controls in cerebrospinal fluid (detection method: OpenArray qPCR).^60^ However, whether these overlapping miRNAs are linked to a shared pathogenesis, or rather a common epiphenomenon linked to a secondary process, such as cell death or neuroinflammation, remains to be elucidated. To gain an insight into the molecular pathology of neurodegeneration in idiopathic Parkinson’s disease and progressive supranuclear palsy, we performed an enriched pathway analysis mapping significantly dysregulated miRNAs and their respective target genes. We identified dysregulated NK cell pathway (dysregulated *miR-197-3p*) to be top ranked both in idiopathic Parkinson’s disease versus controls and progressive supranuclear palsy versus controls. Ten to fifteen per cent of all circulating lymphocytes are NK cells constituting an important part of the innate immune system acting principally via perforin and granzyme to induce cell destruction, antibody-dependent cell cytotoxicity (ADCC) or by inducing apoptosis.^61^ Of note, NK cells were demonstrated to be increased in Parkinson’s disease versus controls^62^ and showed an activated profile in early stages of Parkinson’s disease.^63^ These innate immunity cells have been shown to be implicated in various aging-related diseases via modulation of neuroinflammation and impaired immune homeostasis in the CNS.^64^ In terms of Parkinson’s disease, NK cells were suggested to have a protective role by facilitating the clearance of α-Syn via lysosomal/endosomal degradation, supported by an experimental depletion of NK cells that resulted in increased degeneration of dopaminergic neurons in *substantia nigra* and promoted neuroinflammation in the Parkinson’s disease mouse models.^65^ Equally, a recent study reported alterations in the immune system profile including NK cells CD56^+^ in pathologically confirmed progressive supranuclear palsy patients.^66^ Though the mechanistic understanding for the link between the NK cells and neurodegeneration in Parkinson’s disease and progressive supranuclear palsy remains elusive, our results provide for the first time the evidence from miRNA analyses that the immune system and escalated neuroinflammation are implicated across distinct classes of neurodegenerative disorders. It further corroborates the hypothesis that the immune system may display a more active and cytotoxic state as demonstrated in our recent work on the role of T cells in early- to mid-stage Parkinson’s disease patients.^67^

### Simulation of the significant pathways affected by dysregulated miRNAs

We applied the PBM approach to simulate the effect of significantly dysregulated miRNAs on the affected molecules and related pathways. Both in the idiopathic Parkinson’s disease and progressive supranuclear palsy, we ascertained the dysfunction of the TFEB leading to the lysosomal dysfunction and defective autophagy, i.e. the functions essential for clearing of dysfunctional proteins such as phosphorylated tau or α-Syn fibrils.^68-70^ However, the mechanisms behind the lysosomal dysfunction differed in PBM; whereas in idiopathic Parkinson’s disease, the defective lysosomal acidification was the primary factor (multiple ATP6V molecules involved; see Table 4), in progressive supranuclear palsy, the lysosomal membrane formation was disrupted (CLN3 involved). Our investigation highlighted the pivotal role of ER stress signalling in maintaining cellular integrity, especially under conditions of protein misfolding. This response mechanism is critical in countering the neurotoxic effects of accumulated misfolded proteins, a common feature in Parkinson’s disease and progressive supranuclear palsy.^71^ Based on the PBM simulation of dysregulated miRNAs, we observed more robust autophagic response in progressive supranuclear palsy when compared to idiopathic Parkinson’s disease, especially given the progressive supranuclear palsy–specific perturbation involving ATG7,^72^ while in idiopathic Parkinson’s disease the dysregulated miRNAs involved different molecules like BECN1 and MAP3LC3B. Interestingly, BECN1 was already suggested as a potential therapeutic target in Parkinson’s disease.^73^

In terms of protein homeostasis, progressive supranuclear palsy maintained a stable chaperone-mediated protein folding process in the PBM simulation, whereas idiopathic Parkinson’s disease showed an adaptive upregulation in response mechanisms, particularly through factors like SIRT1 and HSPB1. This adaptive response in Parkinson’s disease underscores a potential therapeutic opportunity through modulation of chaperone activity.^74^ Moreover, ERAD pathway was found to be compromised in both idiopathic Parkinson’s disease and progressive supranuclear palsy. It exhibited a consistent downregulation, particularly associated with SIGMAR1. This was accompanied by the downregulation of ATG7 and increased activity of EI2FK3, exacerbating a potential ERAD phenotype in progressive supranuclear palsy.^75^ Additionally, the differential activity of PPARGC1A in idiopathic Parkinson’s disease and progressive supranuclear palsy might be important to understanding their distinct mitochondrial biogenesis phenotypes. Enhanced PPARGC1A activity was linked to an improved mitochondrial function and was proposed as a therapeutic target, specifically in relation to SIRT3 regulation of mitochondrial biogenesis in aging-related disorders.^76^

In progressive supranuclear palsy, a potential involvement of calcium signalling pathway was supported by the involvement of a range of molecules shown in Table 5. Notably, we observed an inhibition of CREB1 and TORC1 and 2, coupled with the downregulation of NR4A2 and PPARGC1, and a reduction in dopamine receptor endocytic activity. In the idiopathic Parkinson’s disease simulation, the calcium signalling pathway altered downstream CREB signalling, which could affect the neuronal excitotoxicity as indicated in previous studies.^77-81^

In exploring the dopamine transcription pathways and their impact on mitochondrial biogenesis, distinct responses were observed in idiopathic Parkinson’s disease and progressive supranuclear palsy. Progressive supranuclear palsy demonstrated a robust mitochondrial biogenesis, potentially indicative of an adaptive mechanism, as highlighted in previous studies on the AMPK-PGC-1α pathway.^82^ Furthermore, PBM simulation was indicative for a reduced mitochondrial biogenesis. Such observation is supported by research on the critical role of mitochondrial biogenesis in dopamine neuron survival, especially in conditions of PRKN or PINK1 deficiency.^83^ Still focusing on the dopamine transcription pathways, we revealed the DOPA decarboxylase (DDC) to be upregulated in idiopathic Parkinson’s disease. The crucial function of this protein centres around monoamine synthesis and was found to be highly expressed in dopaminergic neurons in *S. nigra.* Interestingly, DDC was identified as a top-hit in proteomic large-scale cross-cohort validation study using CSF of prodromal Parkinson’s disease (i.e. iRBD) versus controls as well as in CSF, plasma and urine in Parkinson’s disease versus controls.^84^ In summary, we demonstrated the potential of *in silico* modelling to unravel putative diagnostic or therapeutic targets using epigenetic markers such as miRNA.

### miRNAs as predictive biomarkers for Parkinson’s disease and progressive supranuclear palsy

In the view of an urgent need for diagnostic biomarkers that would help to accurately discriminate between the neurodegenerative parkinsonism and healthy individuals as well as between the distinct classes of parkinsonian disorders, we inquired into the utility of miRNA(s) as diagnostic biomarkers for Parkinson’s disease and progressive supranuclear palsy. Based on our data set, we reached a comparable AUC to the previous study comparing idiopathic Parkinson’s disease versus controls (model ‘HCvPD’ in Fig. 3: AUC of 0.5/0.66/0.71 including at most 0/10/100 miRNAs versus AUC 0.705 using a panel of 5 brain-enriched miRNAs reported by Ravanidis *et al.*^19^). However, looking closely at the models, we see that when we include the unpenalized age and sex into the prediction model adding at most 0/10/100 miRNAs, the AUC was determined 0.72/0.73/0.76. This means that the high AUC in the model idiopathic Parkinson’s disease versus controls (‘HCvPD’) was mainly due to the age and sex with eventually modest contribution of miRNAs in the discrimination between the groups. Such a consideration was crucial when comparing to former reports. As demonstrated in a large meta-analysis by Guévremont^22^ focusing on diagnostic accuracy across large number of studies, the relatively high sensitivity and specificity (0.82 and 0.80, respectively) of miRNAs subsets as diagnostic biomarkers in Parkinson’s disease versus control might have been accounted to an uneven distribution of sex in the data sets, heterogeneity in ethnic origin of subjects or various diagnostic criteria for Parkinson’s disease across the meta-analysed studies. Here, we have addressed all the mentioned limitations by incorporating age and sex in the prediction models, having subjects overwhelmingly of one ethnic origin (European ancestry) and unified diagnostic criteria with monocentric recruitment of the individuals in the study.

Subsequently, we investigated the prediction power between progressive supranuclear palsy versus idiopathic Parkinson’s disease and found it relatively low (AUC 0.5/0.54/0.56; and when including unpenalized age and sex into the model, AUC 0.63/0.61/0.62). However, we should also consider a potential application of a putative biomarker in clinical practice. Such a diagnostic biomarker would be mainly of essence in the early stage of the progressive parkinsonism where differential diagnosis between Parkinson’s disease and progressive supranuclear palsy remains a challenge. Therefore, we stratified the idiopathic Parkinson’s disease and progressive supranuclear palsy group by defining an early disease stage as ≤ 5 years of disease duration since diagnosis and observed a more accurate prediction in terms of AUC with at most 0/10/100 miRNA and unpenalized age and sex to 0.64/0.77/0.75 (model ‘EPDvEPSP_adj’), a result comparable to that of idiopathic Parkinson’s disease versus controls (see Fig. 3).

### Limitations of the study

The controls in Luxembourg Parkinson's Study were included in the study in the absence of (i) evidence for a neurodegenerative disorder; (ii) active cancer; and (iii) pregnancy. However, other comorbidities (reported or even those not reported or not known) might influence the miRNA profiles in all groups. We addressed this fact by adjusting for effect of age and sex in the differential expression analysis and in the prediction models, but we acknowledge that the frequencies of comorbidities might follow a non-linear trajectory to the age and sex.

Next, we acknowledge that employing the microarray for miRNA detection is less performant compared to other existing detection methods, i.e. RNA sequencing (RNA-seq). Though acknowledging a potential bias in miRNA detection in this study, our previous collaborative work on miRNA using microarray and RNA-seq in >1.000 and >5.000 blood samples from the Luxembourg Parkinson's Study and Parkinson's Progression Markers Initiative (PPMI) showed a very high concordance of significantly dysregulated miRNAs in Parkinson’s disease versus controls.^45^ Hence, we argue that we can largely substantiate our findings presented in this study given the previous replication of the miRNA profiles in Luxembourg Parkinson's Study to PPMI by two independent methods, especially when considering that we address other very important limitations in previous studies on miRNA in Parkinson’s disease and related disorders (very comprehensive genetic screening, exclusion of blood relatives, universally homogeneous ancestry and large sample sizes for all investigated groups).

Furthermore, we observed a higher proportion of family history of Parkinson’s disease or dementia in control group versus idiopathic Parkinson’s disease (27 and 31% versus 25 and 22%, respectively). We explain this phenomenon by the observation that controls supporting research were prone to do so due to the experience with the neurodegenerative disorders in their families and/or are accompanying the patients to the research clinic and joining the study. This limitation was partially addressed by excluding all first–third-degree blood relatives and all Parkinson’s disease–linked mutation carriers identified after a comprehensive genetic screening.

Finally, it is important to stress that the results of the PBM-based simulation refer to the *in silico* modelling, and our results warrant further validation in functional studies.

## Conclusion

Based on our results, we cannot confirm that miRNA panels detected via microarray alone are useful diagnostic biomarkers in idiopathic Parkinson’s disease and progressive supranuclear palsy given the low-to-modest contribution in predictive power of miRNAs. In contrast, our differential expression analysis of miRNAs and the subsequent dysregulated miRNA pathway mapping have provided an insight into the pathophysiological processes manifested during the disease course of idiopathic Parkinson’s disease and progressive supranuclear palsy highlighting a potential implication of immune system in terms of NK cells. Furthermore, we endeavoured to focus on replication of the previously reported dysregulated miRNAs using a large reference meta-analysis rather than *ad hoc* comparisons to the previous underpowered studies. With this approach, we replicated three robustly reported dysregulated miRNAs (*miR-141-3p*, *miR-451a* and *miR-185-5p*) that might warrant further investigations in their role in the pathogenesis and progression of Parkinson’s disease. Finally, we identified significant overlapping miRNAs between idiopathic Parkinson’s disease and progressive supranuclear palsy in comparison to controls that might point to a partially converging nature of pathophysiological processes across distinct classes of neurodegenerative disorders. To further support our hypothesis, we applied the PBM in the Parkinson’s disease map to mechanistically understand the effect of dysregulated miRNAs on the molecular level, and with this approach, we demonstrated partially convergent end-point dysfunction in Parkinson’s disease and progressive supranuclear palsy at the cellular level, though the overlap in end-point dysfunction differed in the dysregulated molecules and genes involved. To conclude, these *in silico* insights might well serve and instruct the development of the future tailored therapies for Parkinson’s disease and progressive supranuclear palsy.

## Supplementary Material

fcae187_Supplementary_Data

## Data Availability

The data set for this manuscript is not publicly available as it is linked to the Luxembourg Parkinson's Study and its internal regulations. Any reasonable requests for accessing the pseudonymized data set can be directed to request.ncer-pd@uni.lu. The code for the statistical models is available at https://doi.org/10.17881/aytm-r037.

## Appendix I

### The members of the NCER-PD Consortium

Geeta Acharya, Gloria Aguayo, Myriam Alexandre, Muhammad Ali, Wim Ammerlann, Rudi Balling, Michele Bassis, Katy Beaumont, Regina Becker, Camille Bellora, Guy Berchem, Daniela Berg, Alexandre Bisdorff, Kathrin Brockmann, Jessica Calmes, Lorieza Castillo, Gessica Contesotto, Giuseppe Arena, Nico Diederich, Rene Dondelinger, Daniela Esteves, Guy Fagherazzi, Jean-Yves Ferrand, Manon Gantenbein, Thomas Gasser, Piotr Gawron, Soumyabrata Ghosh, Marijus Giraitis, Enrico Glaab, Clarissa Gomes, Elisa Gómez De Lope, Jérôme Graas, Mariella Graziano, Valentin Groues, Anne Grünewald, Wei Gu, Gaël Hammot, Anne-Marie Hanff, Linda Hansen, Maxime Hansen, Michael Heneka, Estelle Henry, Sylvia Herbrink, Sascha Herzinger, Michael Heymann, Michele Hu, Alexander Hundt, Ivana Paccoud, Nadine Jacoby, Jacek Jaroslaw Lebioda, Yohan Jaroz, Quentin Klopfenstein, Jochen Klucken, Rejko Krüger, Pauline Lambert, Zied Landoulsi, Roseline Lentz, Inga Liepelt, Robert Liszka, Laura Longhino, Victoria Lorentz, Paula Cristina Lupu, Clare Mackay, Walter Maetzler, Katrin Marcus, Guilherme Marques, Tainá Marques, Patricia Martins Conde, Patrick May, Deborah Mcintyre, Chouaib Mediouni, Francoise Meisch, Myriam Menster, Maura Minelli, Michel Mittelbronn, Brit Mollenhauer, Carlos Moreno, Friedrich Mühlschlegel, Romain Nati, Ulf Nehrbass, Sarah Nickels, Beatrice Nicolai, Jean-Paul Nicolay, Fozia Noor, Marek Ostaszewski, Sinthuja Paccontrolshek, Claire Pauly, Laure Pauly, Lukas Pavelka, Magali Perquin, Rosalina Ramos Lima, Armin Rauschenberger, Rajesh Rawal, Dheeraj Reddy Bobbili, Eduardo Rosales, Isabel Rosety, Kirsten Rump, Estelle Sandt, Stefano Sapienza, Venkata Satagopam, Margaux Schmitt, Sabine Schmitz, Reinhard Schneider, Jens Schwamborn, Jean-Edouard Schweitzer, Amir Sharify, Ekaterina Soboleva, Kate Sokolowska, Olivier Terwindt, Hermann Thien, Elodie Thiry, Rebecca Ting Jiin Loo, Christophe Trefois, Johanna Trouet, Olena Tsurkalenko, Michel Vaillant, Mesele Valenti, Sijmen Van Schagen, Liliana Vilas Boas, Maharshi Vyas, Richard Wade-Martins, Paul Wilmes, Evi Wollscheid-Lengeling and Gelani Zelimkhanov.
