# Supplementary material for: Converging peripheral blood microRNA profiles in Parkinson's disease and progressive supranuclear palsy
Source: Brain Commun. 2024 May 31;6(3):fcae187. doi: 10.1093/braincomms/fcae187 (PMC11166179; doi:10.1093/braincomms/fcae187)
Supplement: fcae187_Supplementary_Data [file fcae187_supplementary_data.zip › Supplementary_material.docx]

**Supplementary material**

**Sensitivity analysis -methods**

The sensitivity analysis was performed to evaluate the impact of molecular perturbations in Boolean models, focusing on how perturbations affect the model's dynamics and stability. This was achieved by assessing the variations in the attractors - the states the model system tends to be stable under different conditions. We applied node perturbations wherein the state of a single biomolecule was altered, either through knockout (complete deactivation) or overexpression (enhanced activity). This approach allowed for the assessment of the individual contribution of each biomolecule to the model's overall function. The effect of these perturbations on the model was closely monitored, specifically focusing on how the alteration of a single node influenced the model's dynamics and stability. In sensitivity analysis, two distinct metrics were performed to effectively quantify the differences between the unperturbed and perturbed attractors, within our models.

The first metric, which we refer to as similarity-based distance, was utilised to assess the overlap and uniqueness in the states of the attractors. This metric allowed us to evaluate the extent of similarity and difference between the two attractors, thus providing insights into the impact of perturbations on the system's state.

$$D\_sim(A, B) = 1 - (\Sigma(min(a\_i, b\_i)) / \Sigma(max(a\_i, b\_i)))$$

- $A, B$ represent the state vectors of the two attractors being compared.
- $a\_i, b\_i$ are the values of the ith state in attractors $A, B$ respectively.
- The numerator calculates the sum of the minimum values of each state between the two attractors.
- The denominator calculates the sum of the maximum values of each state between the two attractors.
- This metric measures the overlap and uniqueness of the attractors. A value close to 0 indicates high similarity, while a value closer to 1 indicates greater difference.

The second metric, known as identity-based distance, was developed to measure the proportion of shared states between the attractors. This helped us understand the degree of change induced by the perturbations. This approach was significant in deciphering the resilience of the network to various perturbations and in identifying critical nodes or interactions that have a significant influence on the network's behaviour.

$D\_id(A, B) = (\Sigma(\delta(a\_i, b\_i))) / n$

- δ(a_i, b_i) is an indicator function, which equals 1 if $a\_i = b\_i$ and 0 otherwise
- This equation sums the number of states that are identical in both attractors and divides by the total number of states $n$
- This metric assesses the proportion of shared states between the attractors, indicating the degree of change induced by perturbations. A higher value implies more shared states and thus less change.

**The members of NCER-PD consortium:**

Geeta ACHARYA ^2^, Gloria AGUAYO ^2^, Myriam ALEXANDRE ^2^, Muhammad ALI ^1^, Wim AMMERLANN ^2^, Rudi BALLING ^1^, Michele BASSIS ^1^, Katy BEAUMONT ^2^, Regina BECKER ^1^, Camille BELLORA ^2^, Guy BERCHEM ^3^, Daniela BERG ^11^, Alexandre BISDORFF ^5^, Kathrin BROCKMANN ^11^, Jessica CALMES ^2^, Lorieza CASTILLO ^2^, Gessica CONTESOTTO ^2^, Giuseppe ARENA^1,^ Nico DIEDERICH ^3^, Rene DONDELINGER ^5^, Daniela ESTEVES ^2^, Guy FAGHERAZZI ^2^, Jean-Yves FERRAND ^2^, Manon GANTENBEIN ^2^, Thomas GASSER ^11^, Piotr GAWRON ^1^, Soumyabrata GHOSH ^1^, Marijus GIRAITIS ^2,3^, Enrico GLAAB ^1^, Clarissa GOMES ^1^, Elisa GÓMEZ DE LOPE ^1^, Jérôme GRAAS ^2^, Mariella GRAZIANO ^17^, Valentin GROUES ^1^, Anne GRÜNEWALD ^1^, Wei GU ^1^, Gaël HAMMOT ^2^, Anne-Marie HANFF ^2^, Linda HANSEN ^1,3^, Maxime HANSEN ^1,3^, Michael HENEKA ^1^, Estelle HENRY ^2^, Sylvia HERBRINK ^6^, Sascha HERZINGER ^1^, Michael HEYMANN ^2^, Michele HU ^8^, Alexander HUNDT ^2^, Ivana PACCOUD ^2^, Nadine JACOBY ^18^, Jacek JAROSLAW LEBIODA ^1^, Yohan JAROZ ^1^, Quentin KLOPFENSTEIN ^1^, Jochen KLUCKEN ^1,2,3^, Rejko KRÜGER ^1,2,3^, Pauline LAMBERT ^2^, Zied LANDOULSI ^1^, Roseline LENTZ ^7^, Inga LIEPELT ^11^, Robert LISZKA ^14^, Laura LONGHINO ^3^, Victoria LORENTZ ^2^, Paula Cristina LUPU ^2^, Clare MACKAY ^10^, Walter MAETZLER ^15^, Katrin MARCUS ^13^, Guilherme MARQUES ^2^, Tainá MARQUES ^1^, Patricia MARTINS CONDE ^1^, Patrick MAY ^1^, Deborah MCINTYRE ^2^, Chouaib MEDIOUNI ^2^, Francoise MEISCH ^1^, Myriam MENSTER ^2^, Maura MINELLI ^2^, Michel MITTELBRONN ^1,4^, Brit MOLLENHAUER ^12^, Carlos MORENO ^1^, Friedrich MÜHLSCHLEGEL ^4^, Romain NATI ^3^, Ulf NEHRBASS ^2^, Sarah NICKELS ^1^, Beatrice NICOLAI ^3^, Jean-Paul NICOLAY ^19^, Fozia NOOR ^2^, Marek OSTASZEWSKI ^1^, Sinthuja PACCONTROLSHEK ^1^, Claire PAULY ^1,3^, Laure PAULY ^1^, Lukas PAVELKA ^1,2,3^, Magali PERQUIN ^2^, Rosalina RAMOS LIMA ^2^, Armin RAUSCHENBERGER ^1^, Rajesh RAWAL ^1^, Dheeraj REDDY BOBBILI ^1^, Eduardo ROSALES ^2^, Isabel ROSETY ^1^, Kirsten RUMP ^1^, Estelle SANDT ^2^, Stefano SAPIENZA ^1^, Venkata SATAGOPAM ^1^, Margaux SCHMITT ^2^, Sabine SCHMITZ ^1^, Reinhard SCHNEIDER ^1^, Jens SCHWAMBORN ^1^, Jean-Edouard SCHWEITZER ^1^, Amir SHARIFY ^2^, Ekaterina SOBOLEVA ^1^, Kate SOKOLOWSKA ^2^, Olivier TERWINDT ^1,3^, Hermann THIEN ^2^, Elodie THIRY ^3^, Rebecca TING JIIN LOO ^1^, Christophe TREFOIS ^1^, Johanna TROUET ^2^, Olena TSURKALENKO ^2^, Michel VAILLANT ^2^, Mesele VALENTI ^2^, Sijmen VAN SCHAGEN ^1^, Liliana VILAS BOAS ^3^, Maharshi VYAS ^1^, Richard WADE-MARTINS ^9^, Paul WILMES ^1^, Evi WOLLSCHEID-LENGELING ^1^, Gelani ZELIMKHANOV ^3^

^1^ Luxembourg Centre for Systems Biomedicine, University of Luxembourg, Esch-sur-Alzette, Luxembourg

^2^ Luxembourg Institute of Health, Strassen, Luxembourg

^3^ Centre Hospitalier de Luxembourg, Strassen, Luxembourg

^4^ Laboratoire National de Santé, Dudelange, Luxembourg

^5^ Centre Hospitalier Emile Mayrisch, Esch-sur-Alzette, Luxembourg

^6^ Centre Hospitalier du Nord, Ettelbrück, Luxembourg

^7^ Parkinson Luxembourg Association, Leudelange, Luxembourg

^8^ Oxford Parkinson's Disease Centre, Nuffield Department of Clinical Neurosciences, University of Oxford, Oxford, UK

^9^ Oxford Parkinson's Disease Centre, Department of Physiology, Anatomy and Genetics, University of Oxford, Oxford, UK

^10^ Oxford Centre for Human Brain Activity, Wellcome Centre for Integrative Neuroimaging, Department of Psychiatry, University of Oxford, Oxford, UK

^11^ Center of Neurology and Hertie Institute for Clinical Brain Research, Department of Neurodegenerative Diseases, University Hospital Tübingen, Tübingen, Germany

^12^ Paracelsus-Elena-Klinik, Kassel, Germany

^13^ Ruhr-University of Bochum, Bochum, Germany

^14^ Westpfalz-Klinikum GmbH, Kaiserslautern, Germany

^15^ Department of Neurology, University Medical Center Schleswig-Holstein, Kiel, Germany

^16^ Department of Neurology Philipps, University Marburg, Marburg, Germany

^17^ Association of Physiotherapists in Parkinson's Disease Europe, Esch-sur-Alzette, Luxembourg

^18^ Private practice, Ettelbruck, Luxembourg

^19^ Private practice, Luxembourg, Luxembourg

**Supplementary Tables**

**Supplementary Table 1.** Results for multiple linear regression analysis (mlr). Log fold changes and *p*-values for effect of sex in the model *HC_sex_mlr* (controls, female:male 182:234) on miRNA expression. No effect implies fold change = 1 and log fold change = 0. Significantly upregulated/downregulated miRNAs are highlighted in red/blue respectively. One asterisk means significant at an FDR of 5% (Benjamini-Hochberg), and three asterisks mean significant at an FWER of 5% (Bonferroni) across all comparisons.

| *miRNA*  *upregulated n=129 downregulated n=94* | Effect size | p-value | Significance |
| --- | --- | --- | --- |
| miR-193b-3p | -0.313 | 5.2e-13 | *** |
| miR-503-5p | 0.340 | 1.7e-12 | *** |
| miR-1255b-5p | 0.256 | 3.7e-12 | *** |
| miR-6073 | 0.603 | 7.5e-12 | *** |
| miR-133b | -0.621 | 1.7e-10 | *** |
| miR-145-5p | -0.336 | 7.6e-10 | *** |
| miR-505-5p | 0.253 | 1.8e-09 | *** |
| miR-103a-3p | 0.227 | 2.1e-09 | *** |
| miR-150-5p | -0.371 | 4.0e-09 | *** |
| miR-200c-3p | 0.187 | 5.1e-09 | *** |
| miR-664a-5p | 0.277 | 5.5e-09 | *** |
| miR-223-3p | -0.242 | 6.0e-09 | *** |
| miR-106b-5p | 0.272 | 1.8e-08 | *** |
| miR-590-5p | 0.633 | 3.1e-08 | *** |
| miR-140-3p | -0.219 | 3.2e-08 | *** |
| miR-4323 | -0.239 | 5.5e-08 | *** |
| miR-4753-5p | 0.133 | 7.9e-08 | *** |
| miR-4737 | 0.399 | 8.3e-08 | *** |
| miR-18b-5p | 0.521 | 8.5e-08 | *** |
| miR-107 | 0.237 | 1.0e-07 | *** |
| miR-758-3p | -0.253 | 1.1e-07 | *** |
| miR-34a-5p | 0.291 | 2.0e-07 | *** |
| miR-326 | -0.354 | 2.1e-07 | *** |
| miR-299-5p | -0.339 | 3.1e-07 | *** |
| miR-6088 | -0.143 | 3.7e-07 | *** |
| miR-542-5p | 0.289 | 3.9e-07 | *** |
| miR-766-3p | -0.251 | 4.7e-07 | *** |
| miR-409-5p | -0.236 | 5.2e-07 | *** |
| miR-936 | 0.180 | 5.3e-07 | *** |
| miR-1273h-5p | 0.160 | 6.3e-07 | *** |
| miR-29b-3p | 0.329 | 7.7e-07 | *** |
| miR-122-5p | 0.279 | 8.1e-07 | *** |
| miR-769-3p | 0.104 | 8.3e-07 | *** |
| miR-130a-3p | 0.228 | 9.4e-07 | *** |
| miR-23b-3p | -0.238 | 1.0e-06 | *** |
| miR-133a-3p | -0.159 | 1.1e-06 | *** |
| miR-130b-3p | 0.164 | 1.1e-06 | *** |
| miR-579-5p | 0.104 | 1.1e-06 | *** |
| miR-654-3p | -0.472 | 1.3e-06 | *** |
| miR-1225-5p | -0.146 | 1.3e-06 | *** |
| miR-338-5p | 0.153 | 1.6e-06 | *** |
| miR-18a-5p | 0.503 | 1.6e-06 | *** |
| miR-3179 | 0.168 | 1.7e-06 | *** |
| miR-892b | 0.233 | 1.9e-06 | *** |
| miR-4742-5p | 0.255 | 2.0e-06 | *** |
| miR-23a-3p | -0.241 | 2.7e-06 | *** |
| miR-4443 | 0.281 | 2.8e-06 | *** |
| miR-409-3p | -0.441 | 2.9e-06 | *** |
| miR-30a-3p | -0.178 | 2.9e-06 | *** |
| miR-1912 | -0.157 | 3.1e-06 | *** |
| miR-181c-3p | -0.216 | 3.9e-06 | *** |
| let-7c-5p | 0.311 | 4.3e-06 | *** |
| miR-29b-2-5p | 0.225 | 4.4e-06 | *** |
| miR-3652 | 0.229 | 4.6e-06 | *** |
| miR-361-5p | -0.204 | 4.7e-06 | *** |
| miR-148a-3p | 0.344 | 4.9e-06 | *** |
| miR-342-3p | -0.214 | 5.3e-06 | *** |
| miR-7975 | 0.199 | 5.7e-06 | *** |
| miR-34b-5p | 0.119 | 5.9e-06 | *** |
| miR-3127-5p | 0.216 | 6.0e-06 | *** |
| miR-1185-1-3p | -0.175 | 6.0e-06 | *** |
| miR-411-3p | -0.123 | 6.3e-06 | *** |
| miR-6780a-5p | 0.236 | 6.6e-06 | *** |
| miR-505-3p | -0.213 | 7.2e-06 | *** |
| miR-4281 | -0.169 | 7.3e-06 | *** |
| miR-3907 | 0.324 | 8.5e-06 | *** |
| miR-1255a | 0.154 | 8.6e-06 | *** |
| miR-3163 | 0.189 | 9.3e-06 | *** |
| miR-6134 | 0.143 | 9.4e-06 | *** |
| miR-1271-5p | -0.202 | 9.7e-06 | *** |
| miR-548aw | 0.117 | 1.1e-05 | *** |
| miR-132-3p | 0.172 | 1.2e-05 | *** |
| miR-199a-5p | -0.337 | 1.3e-05 | *** |
| miR-485-3p | -0.404 | 1.4e-05 | *** |
| miR-6792-5p | 0.144 | 1.4e-05 | *** |
| miR-154-5p | -0.327 | 1.7e-05 | *** |
| miR-3156-5p | 0.166 | 1.8e-05 | *** |
| miR-6821-5p | -0.129 | 2.4e-05 | * |
| miR-134-3p | -0.101 | 2.6e-05 | * |
| miR-6754-5p | 0.102 | 2.9e-05 | * |
| miR-543 | -0.267 | 3.0e-05 | * |
| miR-5581-5p | 0.207 | 3.2e-05 | * |
| miR-4516 | -0.222 | 3.3e-05 | * |
| let-7i-5p | 0.226 | 3.5e-05 | * |
| miR-329-3p | -0.269 | 3.7e-05 | * |
| miR-1288-3p | 0.188 | 3.8e-05 | * |
| miR-127-3p | -0.243 | 3.9e-05 | * |
| miR-6767-5p | 0.181 | 4.0e-05 | * |
| miR-323a-3p | -0.293 | 4.4e-05 | * |
| miR-369-5p | -0.121 | 4.5e-05 | * |
| miR-495-3p | -0.331 | 4.7e-05 | * |
| miR-377-3p | -0.306 | 4.8e-05 | * |
| miR-1295a | 0.133 | 4.8e-05 | * |
| miR-3691-5p | 0.090 | 5.7e-05 | * |
| miR-5088-5p | 0.197 | 6.3e-05 | * |
| miR-940 | -0.140 | 6.3e-05 | * |
| miR-610 | 0.154 | 6.6e-05 | * |
| miR-7-5p | 0.254 | 7.1e-05 | * |
| miR-629-3p | -0.188 | 7.2e-05 | * |
| miR-6090 | -0.152 | 7.4e-05 | * |
| miR-1304-5p | 0.101 | 7.7e-05 | * |
| miR-496 | -0.102 | 7.8e-05 | * |
| miR-208a-5p | 0.114 | 7.9e-05 | * |
| miR-31-5p | -0.195 | 8.5e-05 | * |
| miR-1275 | -0.159 | 8.9e-05 | * |
| miR-6734-5p | 0.186 | 9.5e-05 | * |
| miR-4685-5p | 0.284 | 9.6e-05 | * |
| miR-6856-5p | 0.125 | 9.7e-05 | * |
| miR-5194 | 0.163 | 9.9e-05 | * |
| miR-1305 | 0.178 | 1.1e-04 | * |
| miR-642b-5p | -0.104 | 1.2e-04 | * |
| miR-4716-3p | 0.201 | 1.2e-04 | * |
| miR-378g | 0.238 | 1.2e-04 | * |
| miR-4659b-3p | -0.196 | 1.3e-04 | * |
| miR-3189-5p | 0.105 | 1.3e-04 | * |
| miR-99b-5p | -0.227 | 1.4e-04 | * |
| miR-3125 | 0.192 | 1.6e-04 | * |
| miR-6512-5p | 0.191 | 1.7e-04 | * |
| miR-4514 | 0.117 | 1.7e-04 | * |
| miR-1185-2-3p | -0.110 | 1.9e-04 | * |
| miR-101-3p | 0.423 | 2.0e-04 | * |
| miR-424-5p | 0.376 | 2.2e-04 | * |
| miR-6807-5p | 0.163 | 2.2e-04 | * |
| miR-410-3p | -0.226 | 2.3e-04 | * |
| miR-493-5p | -0.183 | 2.5e-04 | * |
| miR-6794-5p | 0.142 | 2.5e-04 | * |
| miR-3960 | -0.182 | 2.7e-04 | * |
| miR-4513 | 0.229 | 2.8e-04 | * |
| miR-4784 | 0.114 | 3.3e-04 | * |
| let-7b-5p | 0.259 | 3.4e-04 | * |
| miR-21-3p | 0.260 | 3.4e-04 | * |
| miR-3140-3p | 0.111 | 3.5e-04 | * |
| miR-3176 | 0.152 | 3.6e-04 | * |
| miR-139-5p | -0.193 | 3.6e-04 | * |
| miR-4289 | 0.102 | 3.6e-04 | * |
| miR-6720-3p | 0.112 | 4.0e-04 | * |
| miR-4659b-5p | -0.132 | 4.6e-04 | * |
| miR-3135b | 0.257 | 4.6e-04 | * |
| miR-4779 | 0.106 | 4.8e-04 | * |
| miR-5003-5p | 0.124 | 5.0e-04 | * |
| miR-376a-3p | -0.319 | 5.4e-04 | * |
| miR-3120-3p | -0.105 | 5.7e-04 | * |
| miR-3161 | 0.102 | 5.8e-04 | * |
| miR-614 | 0.090 | 5.8e-04 | * |
| miR-660-5p | 0.228 | 6.1e-04 | * |
| miR-579-3p | 0.103 | 6.2e-04 | * |
| miR-6131 | 0.167 | 6.3e-04 | * |
| miR-4713-3p | 0.168 | 6.5e-04 | * |
| miR-29a-3p | -0.178 | 6.8e-04 | * |
| miR-4653-3p | 0.169 | 7.1e-04 | * |
| miR-93-3p | -0.150 | 7.5e-04 | * |
| miR-101-5p | 0.277 | 7.7e-04 | * |
| miR-337-3p | -0.273 | 7.7e-04 | * |
| miR-3190-3p | 0.086 | 8.4e-04 | * |
| miR-381-3p | -0.182 | 8.5e-04 | * |
| miR-330-3p | -0.131 | 9.2e-04 | * |
| miR-433-3p | -0.145 | 9.4e-04 | * |
| miR-340-5p | 0.296 | 9.9e-04 | * |
| miR-376a-5p | -0.180 | 1.0e-03 | * |
| miR-3198 | 0.163 | 1.1e-03 | * |
| let-7e-5p | 0.233 | 1.1e-03 | * |
| miR-7152-3p | 0.151 | 1.2e-03 | * |
| miR-1914-3p | 0.154 | 1.2e-03 | * |
| miR-4466 | -0.112 | 1.2e-03 | * |
| miR-378f | 0.154 | 1.3e-03 | * |
| miR-202-3p | 0.089 | 1.3e-03 | * |
| miR-335-3p | -0.119 | 1.3e-03 | * |
| miR-5581-3p | -0.072 | 1.4e-03 | * |
| miR-193a-3p | 0.210 | 1.4e-03 | * |
| miR-6510-5p | 0.177 | 1.4e-03 | * |
| miR-6800-5p | -0.118 | 1.5e-03 | * |
| miR-668-3p | -0.087 | 1.5e-03 | * |
| miR-4659a-3p | -0.151 | 1.5e-03 | * |
| let-7d-5p | 0.158 | 1.6e-03 | * |
| miR-423-3p | -0.142 | 1.6e-03 | * |
| miR-26a-1-3p | -0.063 | 1.6e-03 | * |
| miR-6717-5p | 0.144 | 1.6e-03 | * |
| miR-129-1-3p | -0.108 | 1.6e-03 | * |
| miR-4657 | 0.085 | 1.6e-03 | * |
| miR-1226-5p | 0.083 | 1.6e-03 | * |
| miR-5193 | -0.065 | 1.7e-03 | * |
| miR-654-5p | -0.090 | 1.7e-03 | * |
| miR-22-5p | 0.175 | 1.7e-03 | * |
| miR-497-5p | 0.117 | 1.7e-03 | * |
| miR-513c-3p | 0.060 | 1.9e-03 | * |
| miR-663a | -0.121 | 2.0e-03 | * |
| miR-1307-5p | 0.101 | 2.0e-03 | * |
| miR-4648 | 0.085 | 2.0e-03 | * |
| miR-4442 | 0.141 | 2.0e-03 | * |
| miR-10b-5p | -0.086 | 2.0e-03 | * |
| miR-1233-5p | 0.171 | 2.1e-03 | * |
| miR-6829-5p | 0.090 | 2.1e-03 | * |
| miR-6757-5p | 0.154 | 2.3e-03 | * |
| miR-3936 | 0.079 | 2.4e-03 | * |
| miR-487b-3p | -0.207 | 2.5e-03 | * |
| miR-6089 | -0.162 | 2.5e-03 | * |
| miR-542-3p | 0.108 | 2.6e-03 | * |
| miR-10a-5p | -0.197 | 2.6e-03 | * |
| miR-548q | 0.118 | 2.7e-03 | * |
| miR-491-5p | -0.097 | 2.7e-03 | * |
| miR-181a-2-3p | -0.122 | 2.8e-03 | * |
| miR-4518 | 0.108 | 2.9e-03 | * |
| miR-5701 | 0.095 | 3.0e-03 | * |
| miR-136-5p | -0.113 | 3.2e-03 | * |
| miR-6130 | -0.113 | 3.3e-03 | * |
| miR-3665 | -0.108 | 3.4e-03 | * |
| miR-1236-5p | 0.107 | 3.4e-03 | * |
| let-7i-3p | 0.082 | 3.5e-03 | * |
| miR-301a-3p | 0.368 | 3.5e-03 | * |
| miR-140-5p | 0.231 | 3.5e-03 | * |
| miR-224-5p | -0.166 | 3.6e-03 | * |
| miR-6879-5p | 0.142 | 3.7e-03 | * |
| miR-4268 | -0.072 | 3.8e-03 | * |
| miR-550a-3p | -0.194 | 3.9e-03 | * |
| miR-4796-5p | -0.060 | 3.9e-03 | * |
| miR-363-5p | 0.081 | 4.0e-03 | * |
| miR-378d | 0.153 | 4.0e-03 | * |
| miR-608 | -0.072 | 4.1e-03 | * |
| miR-4465 | 0.155 | 4.1e-03 | * |
| miR-4684-5p | -0.060 | 4.1e-03 | * |
| miR-3607-3p | 0.090 | 4.2e-03 | * |
| miR-5591-3p | -0.058 | 4.2e-03 | * |
| miR-212-3p | 0.137 | 4.3e-03 | * |

**Supplementary Table 2.** Results for multiple linear regression analysis (mlr). Log fold changes and *p*-values for effect of sex in the model *PD_sex_mlr* (Parkinson’s disease, female:male 116:251) on miRNA expression. No effect implies fold change = 1 and log fold change = 0. Significantly upregulated/downregulated miRNAs are highlighted in red/blue respectively. One asterisk means significant at an FDR of 5% (Benjamini-Hochberg), and three asterisks mean significant at an FWER of 5% (Bonferroni) across all comparisons.

| *miRNA*  *upregulated n=15 downregulated n=34* | Effect size | p-value | Significance |
| --- | --- | --- | --- |
| miR-3940-5p | -0.212 | 3.6e-06 | *** |
| miR-224-5p | -0.282 | 1.3e-05 | *** |
| miR-7-5p | 0.388 | 2.0e-05 | * |
| miR-6789-5p | -0.352 | 2.1e-05 | * |
| miR-193b-3p | -0.214 | 2.2e-05 | * |
| miR-1249-5p | -0.189 | 2.7e-05 | * |
| miR-503-5p | 0.247 | 2.8e-05 | * |
| miR-150-5p | -0.294 | 2.8e-05 | * |
| miR-133a-3p | -0.174 | 2.9e-05 | * |
| miR-130b-3p | 0.184 | 3.0e-05 | * |
| miR-133b | -0.485 | 3.2e-05 | * |
| miR-6779-5p | -0.237 | 5.7e-05 | * |
| miR-625-5p | -0.213 | 6.7e-05 | * |
| miR-664a-5p | 0.255 | 7.3e-05 | * |
| miR-3616-3p | -0.193 | 8.5e-05 | * |
| miR-3665 | -0.177 | 8.7e-05 | * |
| miR-136-3p | -0.136 | 1.1e-04 | * |
| miR-3196 | -0.178 | 1.1e-04 | * |
| miR-4742-5p | 0.235 | 1.1e-04 | * |
| miR-4532 | -0.116 | 1.2e-04 | * |
| miR-1228-5p | 0.210 | 1.4e-04 | * |
| miR-4443 | 0.257 | 1.4e-04 | * |
| miR-129-1-3p | -0.125 | 1.6e-04 | * |
| miR-3656 | -0.268 | 1.9e-04 | * |
| miR-31-5p | -0.204 | 1.9e-04 | * |
| miR-6786-5p | -0.157 | 2.1e-04 | * |
| miR-1912 | -0.134 | 2.1e-04 | * |
| miR-6803-5p | -0.320 | 2.4e-04 | * |
| miR-4665-5p | -0.257 | 3.0e-04 | * |
| miR-744-5p | -0.176 | 3.2e-04 | * |
| miR-34a-5p | 0.235 | 3.2e-04 | * |
| let-7i-5p | 0.242 | 3.5e-04 | * |
| miR-625-3p | -0.163 | 3.9e-04 | * |
| miR-6850-5p | -0.156 | 4.0e-04 | * |
| miR-424-3p | 0.198 | 4.3e-04 | * |
| miR-125b-1-3p | -0.182 | 4.7e-04 | * |
| miR-3188 | -0.105 | 4.9e-04 | * |
| miR-4466 | -0.155 | 5.1e-04 | * |
| miR-1911-3p | 0.085 | 5.3e-04 | * |
| miR-6871-5p | 0.160 | 5.4e-04 | * |
| miR-6852-5p | -0.115 | 5.9e-04 | * |
| miR-664b-5p | 0.388 | 6.1e-04 | * |
| miR-4672 | -0.154 | 6.7e-04 | * |
| miR-145-5p | -0.208 | 6.7e-04 | * |
| miR-802 | -0.117 | 8.3e-04 | * |
| miR-4748 | 0.109 | 8.4e-04 | * |
| miR-6800-5p | -0.146 | 8.5e-04 | * |
| miR-4739 | -0.156 | 9.4e-04 | * |
| miR-6500-3p | 0.109 | 9.5e-04 | * |

**Supplementary Table 3.** Results for multiple linear regression analysis (mlr). Log fold changes and *p*-values for effect of sex in the model *PSP_sex_mlr* (progressive supranuclear palsy, female:male 15:20) on miRNA expression. No effect implies fold change = 1 and log fold change = 0. Significantly upregulated/downregulated miRNAs are highlighted in red/blue respectively. One asterisk means significant at an FDR of 5% (Benjamini-Hochberg), and three asterisks mean significant at an FWER of 5% (Bonferroni) across all comparisons.

| *miRNA*  *upregulated n=0 downregulated n=2* | Effect size | p-value | Significance |
| --- | --- | --- | --- |
| miR-511-3p | -0.497 | 1.1e-05 | *** |
| miR-3688-5p | -0.357 | 1.3e-05 | *** |

**Supplementary Table 4.** Results for multiple linear regression analysis (mlr). Log fold changes and *p*-values for effect of age at assessment (AAA) in the model *HC_AAA_mlr* (controls, n=416) on miRNA expression. No effect implies fold change = 1 and log fold change = 0. Significantly upregulated/downregulated miRNAs are highlighted in red/blue respectively. One asterisk means significant at an FDR of 5% (Benjamini-Hochberg), and three asterisks mean significant at an FWER of 5% (Bonferroni) across all comparisons.

| *miRNA*  *upregulated n=101 downregulated n=106* | Effect size | p-value | Significance |
| --- | --- | --- | --- |
| miR-31-5p | -0.015 | 1.1e-11 | *** |
| miR-196b-5p | -0.022 | 1.6e-10 | *** |
| miR-199a-3p | -0.032 | 9.9e-10 | *** |
| miR-20a-3p | -0.021 | 1.8e-09 | *** |
| miR-20a-5p | -0.023 | 7.9e-09 | *** |
| miR-598-3p | -0.015 | 9.7e-09 | *** |
| miR-17-5p | -0.016 | 1.3e-08 | *** |
| miR-29c-5p | 0.014 | 2.0e-08 | *** |
| miR-454-3p | -0.027 | 2.5e-08 | *** |
| miR-4516 | 0.012 | 6.1e-08 | *** |
| miR-27b-3p | -0.017 | 2.8e-07 | *** |
| miR-16-5p | -0.012 | 5.2e-07 | *** |
| miR-126-3p | -0.024 | 6.0e-07 | *** |
| miR-18a-5p | -0.022 | 6.9e-07 | *** |
| miR-15a-5p | -0.017 | 8.7e-07 | *** |
| miR-17-3p | -0.018 | 1.2e-06 | *** |
| miR-6821-5p | 0.006 | 1.3e-06 | *** |
| miR-20b-5p | -0.017 | 1.4e-06 | *** |
| miR-126-5p | -0.026 | 1.5e-06 | *** |
| miR-144-5p | -0.034 | 1.5e-06 | *** |
| miR-1973 | 0.011 | 1.7e-06 | *** |
| miR-432-5p | -0.008 | 2.0e-06 | *** |
| miR-548am-5p | -0.006 | 2.3e-06 | *** |
| miR-6090 | 0.008 | 3.3e-06 | *** |
| miR-1207-5p | 0.006 | 4.0e-06 | *** |
| miR-379-5p | -0.008 | 4.0e-06 | *** |
| miR-101-3p | -0.022 | 4.3e-06 | *** |
| miR-146b-5p | -0.016 | 4.4e-06 | *** |
| miR-539-5p | -0.006 | 4.6e-06 | *** |
| miR-15b-5p | -0.007 | 4.8e-06 | *** |
| miR-642a-3p | 0.008 | 5.6e-06 | *** |
| miR-643 | -0.006 | 5.9e-06 | *** |
| miR-487b-3p | -0.013 | 6.2e-06 | *** |
| miR-494-3p | 0.015 | 6.4e-06 | *** |
| miR-140-5p | -0.015 | 6.6e-06 | *** |
| miR-4515 | 0.005 | 7.2e-06 | *** |
| miR-548d-5p | -0.005 | 8.8e-06 | *** |
| miR-301a-3p | -0.024 | 9.2e-06 | *** |
| miR-18b-5p | -0.018 | 9.5e-06 | *** |
| miR-1225-5p | 0.006 | 9.7e-06 | *** |
| miR-95-3p | -0.004 | 1.0e-05 | *** |
| miR-195-5p | -0.020 | 1.1e-05 | *** |
| miR-374a-5p | -0.027 | 1.1e-05 | *** |
| miR-144-3p | -0.032 | 1.4e-05 | *** |
| miR-197-5p | 0.008 | 1.5e-05 | *** |
| miR-337-5p | -0.008 | 1.7e-05 | *** |
| miR-493-5p | -0.009 | 1.9e-05 | *** |
| miR-579-3p | -0.005 | 2.0e-05 | * |
| miR-424-5p | -0.019 | 2.1e-05 | * |
| miR-98-5p | -0.017 | 2.3e-05 | * |
| miR-624-5p | -0.016 | 2.3e-05 | * |
| let-7g-5p | -0.016 | 2.4e-05 | * |
| miR-4284 | 0.013 | 2.7e-05 | * |
| miR-3960 | 0.009 | 2.8e-05 | * |
| miR-3162-5p | 0.006 | 2.8e-05 | * |
| miR-32-5p | -0.021 | 2.9e-05 | * |
| miR-26b-5p | -0.020 | 3.0e-05 | * |
| miR-299-5p | -0.012 | 4.1e-05 | * |
| miR-190a-5p | -0.017 | 4.4e-05 | * |
| miR-374b-5p | -0.017 | 4.4e-05 | * |
| miR-1291 | 0.007 | 4.6e-05 | * |
| miR-1202 | 0.006 | 5.3e-05 | * |
| miR-664b-5p | 0.015 | 5.4e-05 | * |
| miR-4485-3p | 0.009 | 5.4e-05 | * |
| miR-4289 | -0.005 | 5.7e-05 | * |
| miR-7641 | 0.011 | 5.8e-05 | * |
| miR-96-5p | -0.018 | 6.0e-05 | * |
| miR-550b-2-5p | -0.008 | 6.2e-05 | * |
| miR-301b-3p | -0.007 | 6.5e-05 | * |
| miR-4485-5p | 0.010 | 7.0e-05 | * |
| miR-4281 | 0.006 | 7.1e-05 | * |
| miR-369-5p | -0.005 | 7.1e-05 | * |
| miR-93-5p | -0.010 | 7.8e-05 | * |
| miR-221-5p | -0.009 | 8.0e-05 | * |
| miR-1181 | 0.005 | 8.3e-05 | * |
| miR-30d-5p | 0.013 | 8.3e-05 | * |
| miR-22-3p | 0.007 | 9.3e-05 | * |
| miR-6749-5p | 0.007 | 1.1e-04 | * |
| miR-92a-3p | 0.005 | 1.2e-04 | * |
| miR-6132 | 0.011 | 1.2e-04 | * |
| miR-376c-3p | -0.013 | 1.4e-04 | * |
| miR-200b-3p | -0.005 | 1.4e-04 | * |
| let-7i-3p | -0.005 | 1.4e-04 | * |
| miR-3136-5p | -0.004 | 1.4e-04 | * |
| miR-192-3p | -0.009 | 1.8e-04 | * |
| let-7f-5p | -0.011 | 1.9e-04 | * |
| miR-325 | -0.004 | 2.0e-04 | * |
| miR-4318 | -0.009 | 2.1e-04 | * |
| miR-431-5p | -0.008 | 2.3e-04 | * |
| miR-6087 | 0.007 | 2.3e-04 | * |
| miR-8063 | 0.007 | 2.5e-04 | * |
| miR-3674 | 0.004 | 2.6e-04 | * |
| miR-3195 | 0.006 | 3.0e-04 | * |
| miR-1290 | 0.006 | 3.2e-04 | * |
| miR-6752-5p | 0.004 | 3.2e-04 | * |
| miR-4739 | 0.006 | 3.4e-04 | * |
| miR-495-3p | -0.012 | 3.5e-04 | * |
| miR-221-3p | -0.008 | 3.5e-04 | * |
| miR-762 | 0.005 | 3.7e-04 | * |
| miR-4651 | 0.004 | 3.7e-04 | * |
| miR-21-5p | -0.010 | 3.8e-04 | * |
| miR-150-3p | 0.005 | 3.8e-04 | * |
| miR-410-3p | -0.009 | 3.9e-04 | * |
| miR-8069 | 0.009 | 4.1e-04 | * |
| miR-148a-3p | -0.011 | 4.4e-04 | * |
| miR-3163 | -0.006 | 4.6e-04 | * |
| miR-19b-1-5p | -0.006 | 4.7e-04 | * |
| let-7a-5p | -0.009 | 4.8e-04 | * |
| miR-7-5p | -0.010 | 4.9e-04 | * |
| miR-4721 | 0.008 | 5.2e-04 | * |
| miR-873-5p | 0.004 | 5.2e-04 | * |
| miR-542-3p | -0.005 | 5.3e-04 | * |
| miR-361-5p | 0.007 | 5.6e-04 | * |
| miR-4728-5p | 0.009 | 5.7e-04 | * |
| miR-7846-3p | 0.009 | 5.9e-04 | * |
| miR-662 | 0.004 | 5.9e-04 | * |
| miR-6127 | 0.008 | 6.0e-04 | * |
| miR-654-3p | -0.014 | 6.1e-04 | * |
| let-7d-5p | -0.007 | 6.1e-04 | * |
| miR-620 | -0.006 | 6.2e-04 | * |
| miR-4634 | 0.006 | 6.4e-04 | * |
| let-7i-5p | -0.008 | 6.5e-04 | * |
| miR-1246 | 0.013 | 7.0e-04 | * |
| miR-34a-5p | 0.008 | 7.4e-04 | * |
| miR-4532 | 0.004 | 7.5e-04 | * |
| miR-1273g-3p | 0.010 | 7.7e-04 | * |
| miR-140-3p | 0.006 | 8.0e-04 | * |
| miR-6875-5p | 0.008 | 8.2e-04 | * |
| miR-141-3p | -0.005 | 8.3e-04 | * |
| miR-4763-3p | 0.005 | 8.4e-04 | * |
| miR-6850-5p | 0.005 | 8.5e-04 | * |
| miR-106b-3p | -0.004 | 9.2e-04 | * |
| miR-4327 | 0.004 | 9.5e-04 | * |
| miR-6826-5p | 0.007 | 9.6e-04 | * |
| miR-6800-5p | 0.005 | 9.7e-04 | * |
| miR-1827 | -0.007 | 9.7e-04 | * |
| miR-25-3p | 0.004 | 9.7e-04 | * |
| miR-6880-5p | 0.005 | 1.0e-03 | * |
| miR-2276-3p | 0.003 | 1.0e-03 | * |
| miR-6728-5p | 0.005 | 1.1e-03 | * |
| miR-425-5p | 0.006 | 1.1e-03 | * |
| miR-3651 | 0.007 | 1.1e-03 | * |
| miR-4306 | 0.005 | 1.2e-03 | * |
| miR-551b-3p | -0.003 | 1.2e-03 | * |
| miR-4428 | 0.007 | 1.2e-03 | * |
| miR-509-3-5p | 0.003 | 1.2e-03 | * |
| miR-3663-3p | 0.005 | 1.3e-03 | * |
| miR-6088 | 0.004 | 1.3e-03 | * |
| miR-23a-3p | 0.007 | 1.3e-03 | * |
| miR-30b-3p | -0.004 | 1.3e-03 | * |
| miR-921 | 0.004 | 1.3e-03 | * |
| miR-660-5p | -0.009 | 1.3e-03 | * |
| miR-4655-3p | 0.012 | 1.4e-03 | * |
| miR-377-3p | -0.010 | 1.4e-03 | * |
| miR-5581-3p | 0.003 | 1.4e-03 | * |
| miR-4697-5p | 0.013 | 1.4e-03 | * |
| miR-5006-5p | 0.006 | 1.4e-03 | * |
| miR-151a-3p | 0.008 | 1.6e-03 | * |
| miR-4500 | -0.005 | 1.6e-03 | * |
| miR-6818-5p | -0.003 | 1.6e-03 | * |
| miR-337-3p | -0.011 | 1.6e-03 | * |
| miR-4999-5p | -0.004 | 1.6e-03 | * |
| miR-6865-5p | 0.004 | 1.6e-03 | * |
| miR-3137 | 0.005 | 1.7e-03 | * |
| miR-6894-5p | 0.004 | 1.7e-03 | * |
| miR-324-3p | 0.006 | 1.7e-03 | * |
| let-7e-5p | -0.010 | 1.7e-03 | * |
| miR-450a-5p | -0.005 | 1.8e-03 | * |
| miR-4742-5p | -0.007 | 1.8e-03 | * |
| miR-193b-3p | -0.006 | 1.9e-03 | * |
| miR-4746-3p | 0.004 | 2.1e-03 | * |
| miR-3648 | 0.004 | 2.1e-03 | * |
| miR-374c-5p | -0.012 | 2.1e-03 | * |
| miR-628-5p | -0.006 | 2.1e-03 | * |
| miR-365a-3p | 0.007 | 2.2e-03 | * |
| miR-6738-5p | 0.004 | 2.3e-03 | * |
| miR-590-5p | -0.015 | 2.3e-03 | * |
| miR-6872-5p | 0.004 | 2.3e-03 | * |
| miR-513a-5p | 0.007 | 2.4e-03 | * |
| miR-329-3p | -0.008 | 2.4e-03 | * |
| miR-1229-5p | 0.005 | 2.5e-03 | * |
| miR-6780b-5p | 0.006 | 2.5e-03 | * |
| miR-4798-5p | -0.003 | 2.6e-03 | * |
| miR-3120-3p | -0.004 | 2.6e-03 | * |
| miR-500a-5p | 0.005 | 2.6e-03 | * |
| miR-576-3p | -0.005 | 2.6e-03 | * |
| miR-3909 | 0.006 | 2.6e-03 | * |
| miR-3923 | -0.003 | 2.7e-03 | * |
| miR-3650 | -0.003 | 2.7e-03 | * |
| miR-4286 | 0.008 | 2.9e-03 | * |
| miR-3654 | 0.004 | 3.0e-03 | * |
| miR-6806-5p | 0.004 | 3.1e-03 | * |
| miR-376a-3p | -0.012 | 3.2e-03 | * |
| miR-758-3p | -0.006 | 3.2e-03 | * |
| miR-4796-3p | -0.003 | 3.2e-03 | * |
| miR-1537-3p | -0.003 | 3.3e-03 | * |
| miR-582-5p | -0.009 | 3.4e-03 | * |
| miR-6747-5p | 0.005 | 3.4e-03 | * |
| miR-5585-3p | 0.006 | 3.5e-03 | * |
| miR-212-5p | 0.002 | 3.5e-03 | * |
| miR-1250-3p | -0.003 | 3.7e-03 | * |
| miR-128-1-5p | 0.004 | 3.7e-03 | * |
| miR-4656 | 0.005 | 3.7e-03 | * |
| miR-6768-5p | 0.004 | 3.9e-03 | * |
| miR-4466 | 0.004 | 3.9e-03 | * |
| miR-665 | 0.006 | 4.0e-03 | * |
| miR-6089 | 0.007 | 4.0e-03 | * |

**Supplementary Table 5.** Results for multiple linear regression analysis (mlr). Log fold changes and *p*-values for effect of age at assessment (AAA) in the model *PD_AAA_mlr* (Parkinson’s disease, n=367) on miRNA expression. No effect implies fold change = 1 and log fold change = 0. Significantly upregulated/downregulated miRNAs are highlighted in red/blue respectively. One asterisk means significant at an FDR of 5% (Benjamini-Hochberg), and three asterisks mean significant at an FWER of 5% (Bonferroni) across all comparisons.

| *miRNA*  *upregulated n=54 downregulated n=47* | Effect size | p-value | Significance |
| --- | --- | --- | --- |
| miR-665 | 0.012 | 2.1e-09 | *** |
| miR-22-3p | 0.011 | 7.8e-09 | *** |
| miR-31-5p | -0.013 | 3.9e-08 | *** |
| miR-20a-3p | -0.022 | 1.5e-07 | *** |
| let-7d-5p | -0.012 | 2.2e-07 | *** |
| miR-199a-3p | -0.029 | 7.8e-07 | *** |
| miR-196b-5p | -0.020 | 7.8e-07 | *** |
| miR-6073 | -0.022 | 8.3e-07 | *** |
| miR-93-5p | -0.015 | 1.2e-06 | *** |
| let-7e-5p | -0.015 | 1.2e-06 | *** |
| miR-20a-5p | -0.023 | 1.2e-06 | *** |
| miR-25-3p | 0.007 | 1.7e-06 | *** |
| miR-126-3p | -0.025 | 2.6e-06 | *** |
| miR-3135b | -0.019 | 3.2e-06 | *** |
| miR-20b-5p | -0.020 | 3.4e-06 | *** |
| miR-454-3p | -0.026 | 3.8e-06 | *** |
| miR-140-3p | 0.008 | 4.6e-06 | *** |
| miR-29c-5p | 0.013 | 6.5e-06 | *** |
| miR-361-5p | 0.010 | 9.3e-06 | *** |
| miR-98-5p | -0.018 | 9.5e-06 | *** |
| miR-30d-5p | 0.017 | 1.0e-05 | *** |
| miR-642a-3p | 0.008 | 1.2e-05 | *** |
| miR-126-5p | -0.024 | 1.9e-05 | *** |
| miR-29b-2-5p | -0.009 | 2.2e-05 | * |
| miR-584-3p | 0.008 | 2.3e-05 | * |
| miR-17-5p | -0.015 | 2.4e-05 | * |
| miR-550a-3p | 0.013 | 2.6e-05 | * |
| miR-2115-5p | 0.010 | 4.1e-05 | * |
| miR-144-5p | -0.033 | 4.9e-05 | * |
| miR-1275 | -0.009 | 5.4e-05 | * |
| miR-769-5p | 0.009 | 5.8e-05 | * |
| miR-378a-5p | 0.013 | 5.9e-05 | * |
| miR-30a-5p | 0.016 | 7.0e-05 | * |
| miR-130a-3p | -0.011 | 7.3e-05 | * |
| miR-34a-5p | 0.011 | 7.4e-05 | * |
| miR-151a-3p | 0.012 | 8.0e-05 | * |
| let-7a-5p | -0.010 | 8.5e-05 | * |
| miR-16-5p | -0.011 | 9.0e-05 | * |
| miR-185-5p | 0.007 | 9.4e-05 | * |
| miR-582-3p | 0.007 | 9.5e-05 | * |
| miR-26b-5p | -0.020 | 9.9e-05 | * |
| miR-181c-5p | -0.010 | 1.0e-04 | * |
| miR-2681-5p | 0.007 | 1.0e-04 | * |
| miR-301a-3p | -0.025 | 1.0e-04 | * |
| miR-29a-3p | 0.011 | 1.1e-04 | * |
| miR-659-3p | 0.006 | 1.1e-04 | * |
| miR-3180-3p | 0.013 | 1.1e-04 | * |
| miR-221-3p | -0.011 | 1.2e-04 | * |
| miR-548q | -0.007 | 1.3e-04 | * |
| miR-1225-5p | 0.006 | 1.3e-04 | * |
| miR-32-5p | -0.019 | 1.4e-04 | * |
| miR-30c-5p | 0.013 | 1.8e-04 | * |
| miR-7641 | 0.011 | 1.9e-04 | * |
| miR-15b-5p | -0.006 | 2.2e-04 | * |
| miR-4270 | 0.008 | 2.2e-04 | * |
| let-7f-5p | -0.011 | 2.4e-04 | * |
| miR-210-3p | 0.009 | 2.5e-04 | * |
| miR-29b-1-5p | -0.009 | 3.1e-04 | * |
| miR-4500 | -0.007 | 3.2e-04 | * |
| miR-505-3p | 0.009 | 3.3e-04 | * |
| miR-589-3p | 0.007 | 3.7e-04 | * |
| miR-502-3p | 0.007 | 3.9e-04 | * |
| miR-4306 | 0.006 | 4.0e-04 | * |
| miR-3610 | 0.006 | 4.2e-04 | * |
| miR-101-3p | -0.020 | 4.6e-04 | * |
| miR-148a-5p | 0.006 | 4.7e-04 | * |
| miR-548d-5p | -0.005 | 4.9e-04 | * |
| miR-4788 | 0.012 | 5.3e-04 | * |
| miR-6749-5p | 0.008 | 5.5e-04 | * |
| miR-18a-5p | -0.020 | 5.8e-04 | * |
| let-7i-5p | -0.010 | 5.8e-04 | * |
| miR-23a-3p | 0.010 | 5.9e-04 | * |
| miR-500a-3p | 0.006 | 6.4e-04 | * |
| miR-659-5p | 0.005 | 6.4e-04 | * |
| miR-425-5p | 0.007 | 7.4e-04 | * |
| miR-7847-3p | 0.008 | 7.5e-04 | * |
| miR-221-5p | -0.008 | 7.8e-04 | * |
| let-7g-5p | -0.013 | 7.8e-04 | * |
| miR-18a-3p | 0.010 | 7.9e-04 | * |
| miR-7152-3p | 0.008 | 8.0e-04 | * |
| miR-500a-5p | 0.007 | 8.3e-04 | * |
| miR-15a-5p | -0.013 | 8.3e-04 | * |
| miR-548am-5p | -0.005 | 8.5e-04 | * |
| miR-190a-5p | -0.014 | 8.8e-04 | * |
| miR-27b-3p | -0.013 | 9.4e-04 | * |
| miR-5787 | 0.008 | 1.0e-03 | * |
| miR-128-3p | 0.009 | 1.1e-03 | * |
| miR-1273g-3p | 0.011 | 1.2e-03 | * |
| miR-139-3p | -0.006 | 1.2e-03 | * |
| miR-493-5p | -0.007 | 1.2e-03 | * |
| miR-194-5p | 0.011 | 1.2e-03 | * |
| miR-4687-3p | 0.005 | 1.3e-03 | * |
| miR-144-3p | -0.028 | 1.3e-03 | * |
| miR-339-3p | 0.008 | 1.4e-03 | * |
| miR-3124-3p | 0.005 | 1.5e-03 | * |
| miR-4685-5p | 0.011 | 1.6e-03 | * |
| miR-103a-2-5p | 0.009 | 1.7e-03 | * |
| miR-28-3p | 0.006 | 1.8e-03 | * |
| miR-6826-5p | 0.009 | 1.8e-03 | * |
| miR-550b-25p | -0.007 | 1.9e-03 | * |
| miR-497-3p | -0.004 | 2.0e-03 | * |

**Supplementary Table 6:** Jaccard index. Size of the intersection divided by size of the union of the nominally significant miRNAs from two comparisons (%). A Jaccard index of 100% means that the two comparisons detect the same set of nominally significant miRNAs (irrespective of the number). A Jaccard index of 50% means that half or the miRNAs that are nominally significant in any of the two comparisons are nominally significant in both comparisons (irrespective of the total number). Jaccard indices above 20% are highlighted in bold. Abbreviations: PD (Parkinson’s disease); PSP (progressive supranuclear palsy); HC (controls); mlr (multiple linear regression); AAA (age at assessment).

|  | HC PD | HC PSP | PD PSP | HC sex | PD sex | PSP sex | HC AAA | PD AAA | PSP AAA | HCvPD.mlr | HCvPSP.mlr | PDvPSP.mlr | HC sex.mlr | PD sex.mlr | PSP sex.mlr | HC AAA.mlr | PD AAA.mlr | PSP AAA.mlr |
| --- | --- | --- | --- | --- | --- | --- | --- | --- | --- | --- | --- | --- | --- | --- | --- | --- | --- | --- |
| HC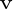PD | **100** | **28** | 5 | 17 | 12 | 8 | **22** | 20 | 3 | **52** | **20** | 4 | 18 | 11 | 7 | **22** | 19 | 3 |
| HC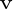PSP | **28** | **100** | **30** | 19 | 10 | 7 | 14 | 16 | 3 | **27** | **61** | **26** | 20 | 9 | 7 | 14 | 16 | 3 |
| PD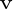PSP | 5 | **30** | **100** | 11 | 5 | 5 | 6 | 6 | 3 | 7 | **34** | **74** | 11 | 5 | 5 | 6 | 6 | 3 |
| HC sex | 17 | 19 | 11 | **100** | 17 | 12 | 19 | 16 | 3 | 16 | 18 | 9 | **92** | 17 | 12 | 19 | 16 | 4 |
| PD sex | 12 | 10 | 5 | 17 | **100** | 10 | 12 | 8 | 3 | 10 | 9 | 5 | 17 | **89** | 10 | 12 | 8 | 4 |
| PSP sex | 8 | 7 | 5 | 12 | 10 | **100** | 16 | 6 | 2 | 6 | 8 | 5 | 12 | 10 | **94** | 15 | 5 | 4 |
| HC AAA | **22** | 14 | 6 | 19 | 12 | 16 | **100** | **22** | 3 | 12 | 11 | 6 | 20 | 12 | 16 | **94** | **22** | 4 |
| PD AAA | 20 | 16 | 6 | 16 | 8 | 6 | **22** | **100** | 2 | 14 | 10 | 4 | 17 | 8 | 6 | **22** | **93** | 2 |
| PSP AAA | 3 | 3 | 3 | 3 | 3 | 2 | 3 | 2 | **100** | 3 | 2 | 3 | 3 | 3 | 3 | 3 | 1 | **85** |
| HC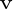PD.mlr | **52** | **27** | 7 | 16 | 10 | 6 | 12 | 14 | 3 | **100** | **22** | 6 | 17 | 9 | 6 | 12 | 14 | 3 |
| HC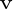PSP.mlr | **20** | **61** | **34** | 18 | 9 | 8 | 11 | 10 | 2 | **22** | **100** | **30** | 18 | 8 | 8 | 11 | 9 | 3 |
| PD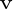PSP.mlr | 4 | **26** | **74** | 9 | 5 | 5 | 6 | 4 | 3 | 6 | **30** | **100** | 9 | 5 | 5 | 6 | 4 | 4 |
| HC sex.mlr | 18 | 20 | 11 | **92** | 17 | 12 | 20 | 17 | 3 | 17 | 18 | 9 | **100** | 17 | 12 | 19 | 17 | 4 |
| PD sex.mlr | 11 | 9 | 5 | 17 | **89** | 10 | 12 | 8 | 3 | 9 | 8 | 5 | 17 | **100** | 10 | 12 | 8 | 4 |
| PSP sex.mlr | 7 | 7 | 5 | 12 | 10 | **94** | 16 | 6 | 3 | 6 | 8 | 5 | 12 | 10 | **100** | 15 | 5 | 5 |
| HC AAA.mlr | **22** | 14 | 6 | 19 | 12 | 15 | **94** | **22** | 3 | 12 | 11 | 6 | 19 | 12 | 15 | **100** | **22** | 4 |
| PD AAA.mlr | 19 | 16 | 6 | 16 | 8 | 5 | **22** | **93** | 1 | 14 | 9 | 4 | 17 | 8 | 5 | **22** | **100** | 2 |
| PSP AAA.mlr | 3 | 3 | 3 | 4 | 4 | 4 | 4 | 2 | **85** | 3 | 3 | 4 | 4 | 4 | 5 | 4 | 2 | **100** |

**Supplementary Table 7.** Comparison HCvPD : Log fold changes and p-values for Parkinson’s disease (PD, n=367) vs. controls (HC, n=416). No effect implies fold change = 1 and log fold change = 0. Significantly upregulated/downregulated miRNAs are highlighted in red/blue respectively. One asterisk means significant at an FDR of 5% (Benjamini-Hochberg), and three asterisks mean significant at an FWER of 5% (Bonferroni) across all comparisons.

| *miRNA*  *upregulated n=126 downregulated n=117* | Effect size | p-value | Significance |
| --- | --- | --- | --- |
| miR-31-5p | -0.265 | 3.9e-12 | *** |
| miR-151a-3p | 0.303 | 6.0e-11 | *** |
| miR-4516 | 0.248 | 9.0e-10 | *** |
| miR-29c-5p | 0.273 | 9.4e-10 | *** |
| miR-20b-5p | -0.391 | 2.0e-09 | *** |
| miR-18a-5p | -0.492 | 5.0e-09 | *** |
| miR-130a-3p | -0.223 | 5.3e-09 | *** |
| miR-145-5p | 0.234 | 7.2e-09 | *** |
| miR-20a-5p | -0.410 | 1.2e-08 | *** |
| miR-22-3p | 0.169 | 1.3e-08 | *** |
| miR-18b-5p | -0.441 | 1.7e-08 | *** |
| miR-1225-5p | 0.130 | 2.6e-08 | *** |
| miR-17-5p | -0.282 | 3.7e-08 | *** |
| miR-378a-5p | 0.264 | 5.6e-08 | *** |
| miR-505-3p | 0.198 | 7.4e-08 | *** |
| miR-30d-5p | 0.303 | 1.8e-07 | *** |
| miR-2115-5p | 0.177 | 3.0e-07 | *** |
| miR-93-5p | -0.231 | 3.8e-07 | *** |
| miR-23a-3p | 0.203 | 5.6e-07 | *** |
| miR-3923 | -0.102 | 9.6e-07 | *** |
| miR-4281 | 0.140 | 1.2e-06 | *** |
| miR-186-5p | 0.233 | 1.2e-06 | *** |
| miR-25-3p | 0.113 | 1.3e-06 | *** |
| miR-126-3p | -0.402 | 1.4e-06 | *** |
| miR-454-3p | -0.405 | 2.2e-06 | *** |
| miR-16-5p | -0.201 | 2.3e-06 | *** |
| miR-491-5p | 0.114 | 2.5e-06 | *** |
| miR-196b-5p | -0.282 | 3.1e-06 | *** |
| miR-374a-5p | -0.511 | 3.5e-06 | *** |
| miR-32-5p | -0.385 | 4.0e-06 | *** |
| let-7i-3p | -0.095 | 4.4e-06 | *** |
| miR-197-3p | 0.186 | 4.5e-06 | *** |
| miR-199a-3p | -0.416 | 4.9e-06 | *** |
| miR-590-5p | -0.406 | 5.3e-06 | *** |
| miR-6749-5p | 0.159 | 5.8e-06 | *** |
| miR-92a-3p | 0.112 | 6.1e-06 | *** |
| miR-103a-3p | -0.130 | 6.5e-06 | *** |
| miR-4286 | 0.207 | 6.7e-06 | *** |
| miR-374b-5p | -0.350 | 8.1e-06 | *** |
| miR-4328 | 0.067 | 1.0e-05 | *** |
| miR-122-5p | -0.173 | 1.1e-05 | *** |
| miR-484 | 0.225 | 1.4e-05 | *** |
| miR-378i | 0.131 | 1.4e-05 | *** |
| miR-660-5p | -0.236 | 1.4e-05 | *** |
| miR-141-3p | -0.118 | 1.5e-05 | *** |
| miR-1202 | 0.108 | 1.7e-05 | *** |
| miR-4289 | -0.090 | 2.0e-05 | * |
| miR-1827 | -0.142 | 2.3e-05 | * |
| miR-98-5p | -0.279 | 2.5e-05 | * |
| miR-664b-3p | 0.127 | 2.6e-05 | * |
| miR-6088 | 0.088 | 2.8e-05 | * |
| miR-3163 | -0.140 | 2.9e-05 | * |
| miR-4500 | -0.117 | 3.3e-05 | * |
| miR-140-5p | -0.256 | 3.3e-05 | * |
| let-7d-5p | -0.153 | 3.5e-05 | * |
| miR-6090 | 0.122 | 3.6e-05 | * |
| miR-1291 | 0.126 | 4.7e-05 | * |
| miR-151b | 0.122 | 4.9e-05 | * |
| miR-624-5p | -0.291 | 4.9e-05 | * |
| miR-3677-5p | 0.144 | 5.1e-05 | * |
| miR-331-3p | 0.178 | 5.2e-05 | * |
| miR-598-3p | -0.186 | 5.4e-05 | * |
| miR-6799-5p | -0.152 | 5.6e-05 | * |
| miR-7106-5p | -0.172 | 5.7e-05 | * |
| miR-665 | 0.128 | 6.0e-05 | * |
| let-7f-5p | -0.188 | 6.2e-05 | * |
| miR-3155a | 0.069 | 7.0e-05 | * |
| miR-374c-5p | -0.276 | 7.0e-05 | * |
| miR-378a-3p | 0.118 | 7.1e-05 | * |
| miR-769-5p | 0.125 | 7.1e-05 | * |
| miR-3136-5p | -0.083 | 7.1e-05 | * |
| miR-4486 | -0.151 | 7.3e-05 | * |
| miR-6720-5p | 0.135 | 7.6e-05 | * |
| miR-1304-5p | -0.079 | 8.5e-05 | * |
| miR-151a-5p | 0.116 | 9.0e-05 | * |
| let-7e-5p | -0.201 | 9.0e-05 | * |
| miR-6770-3p | 0.103 | 9.1e-05 | * |
| miR-301a-3p | -0.378 | 9.1e-05 | * |
| miR-126-5p | -0.347 | 9.5e-05 | * |
| miR-4538 | -0.094 | 1.0e-04 | * |
| let-7d-3p | 0.149 | 1.1e-04 | * |
| miR-6849-5p | -0.140 | 1.1e-04 | * |
| miR-145-3p | 0.058 | 1.1e-04 | * |
| miR-101-3p | -0.336 | 1.2e-04 | * |
| let-7a-5p | -0.155 | 1.3e-04 | * |
| miR-361-5p | 0.130 | 1.3e-04 | * |
| miR-3145-5p | 0.065 | 1.4e-04 | * |
| miR-5090 | -0.080 | 1.4e-04 | * |
| miR-107 | -0.125 | 1.4e-04 | * |
| miR-873-5p | 0.068 | 1.4e-04 | * |
| miR-7641 | 0.174 | 1.5e-04 | * |
| miR-6727-5p | -0.123 | 1.7e-04 | * |
| miR-223-3p | 0.113 | 1.8e-04 | * |
| miR-1285-5p | 0.078 | 1.8e-04 | * |
| miR-193a-5p | 0.115 | 1.8e-04 | * |
| miR-30a-5p | 0.231 | 1.9e-04 | * |
| miR-3140-3p | -0.084 | 2.1e-04 | * |
| let-7g-5p | -0.233 | 2.1e-04 | * |
| miR-20a-3p | -0.231 | 2.3e-04 | * |
| miR-328-3p | 0.159 | 2.4e-04 | * |
| miR-6511b-5p | -0.073 | 2.5e-04 | * |
| miR-30c-5p | 0.200 | 2.5e-04 | * |
| miR-9-5p | -0.062 | 2.6e-04 | * |
| miR-664b-5p | 0.247 | 2.7e-04 | * |
| miR-15a-5p | -0.220 | 2.9e-04 | * |
| miR-579-3p | -0.081 | 3.0e-04 | * |
| miR-331-5p | 0.089 | 3.0e-04 | * |
| miR-501-5p | 0.116 | 3.0e-04 | * |
| miR-324-3p | 0.125 | 3.2e-04 | * |
| miR-6877-3p | 0.079 | 3.4e-04 | * |
| miR-3614-5p | 0.071 | 3.5e-04 | * |
| miR-224-5p | -0.149 | 3.6e-04 | * |
| miR-6716-5p | 0.143 | 3.8e-04 | * |
| miR-4685-3p | 0.059 | 3.9e-04 | * |
| miR-326 | 0.180 | 3.9e-04 | * |
| miR-3651 | 0.139 | 4.0e-04 | * |
| miR-148a-3p | -0.213 | 4.1e-04 | * |
| miR-200b-3p | -0.087 | 4.2e-04 | * |
| miR-6076 | 0.081 | 4.4e-04 | * |
| miR-138-1-3p | 0.084 | 4.4e-04 | * |
| miR-96-5p | -0.267 | 4.4e-04 | * |
| miR-940 | 0.095 | 4.5e-04 | * |
| miR-374b-3p | -0.059 | 4.6e-04 | * |
| miR-4687-5p | 0.070 | 4.9e-04 | * |
| miR-6854-3p | 0.056 | 4.9e-04 | * |
| miR-545-3p | -0.119 | 5.2e-04 | * |
| miR-146b-5p | -0.212 | 5.5e-04 | * |
| miR-125a-5p | 0.164 | 5.7e-04 | * |
| miR-4707-5p | -0.074 | 5.9e-04 | * |
| miR-487b-3p | -0.178 | 6.0e-04 | * |
| miR-937-5p | -0.082 | 7.2e-04 | * |
| miR-6503-3p | 0.078 | 7.5e-04 | * |
| miR-4665-3p | 0.085 | 7.8e-04 | * |
| miR-181b-5p | 0.102 | 7.8e-04 | * |
| miR-500a-5p | 0.102 | 8.1e-04 | * |
| miR-144-5p | -0.413 | 8.5e-04 | * |
| miR-517c-3p | -0.053 | 8.6e-04 | * |
| miR-138-2-3p | -0.097 | 9.5e-04 | * |
| miR-539-3p | 0.051 | 9.6e-04 | * |
| miR-6889-3p | 0.073 | 1.0e-03 | * |
| miR-6885-5p | 0.063 | 1.1e-03 | * |
| miR-548q | -0.094 | 1.1e-03 | * |
| miR-34c-3p | 0.071 | 1.1e-03 | * |
| miR-4515 | 0.067 | 1.1e-03 | * |
| miR-1292-5p | 0.075 | 1.1e-03 | * |
| miR-493-5p | -0.119 | 1.1e-03 | * |
| miR-6802-5p | 0.082 | 1.1e-03 | * |
| miR-6779-5p | -0.118 | 1.1e-03 | * |
| miR-4999-5p | -0.069 | 1.1e-03 | * |
| miR-6081 | 0.093 | 1.1e-03 | * |
| miR-195-5p | -0.262 | 1.2e-03 | * |
| miR-330-3p | 0.094 | 1.2e-03 | * |
| miR-6865-3p | 0.072 | 1.2e-03 | * |
| miR-6789-5p | -0.163 | 1.3e-03 | * |
| miR-6073 | -0.217 | 1.3e-03 | * |
| miR-451a | -0.015 | 1.3e-03 | * |
| miR-365a-3p | 0.128 | 1.3e-03 | * |
| miR-140-3p | 0.093 | 1.4e-03 | * |
| miR-548w | -0.050 | 1.5e-03 | * |
| miR-8064 | -0.075 | 1.5e-03 | * |
| miR-6821-5p | 0.076 | 1.6e-03 | * |
| miR-550b-2-5p | -0.107 | 1.6e-03 | * |
| miR-1294 | -0.063 | 1.6e-03 | * |
| miR-6763-3p | 0.059 | 1.6e-03 | * |
| miR-27b-3p | -0.186 | 1.6e-03 | * |
| miR-26b-5p | -0.254 | 1.7e-03 | * |
| miR-432-5p | -0.095 | 1.7e-03 | * |
| miR-4772-3p | 0.069 | 1.7e-03 | * |
| miR-662 | -0.058 | 1.7e-03 | * |
| miR-1246 | 0.225 | 1.7e-03 | * |
| miR-4638-3p | 0.122 | 1.8e-03 | * |
| miR-6780b-5p | 0.100 | 1.8e-03 | * |
| miR-3690 | 0.077 | 1.9e-03 | * |
| miR-6819-3p | 0.078 | 1.9e-03 | * |
| miR-4723-3p | 0.065 | 1.9e-03 | * |
| miR-890 | 0.069 | 1.9e-03 | * |
| miR-6816-5p | 0.143 | 2.0e-03 | * |
| miR-8068 | -0.073 | 2.0e-03 | * |
| miR-28-3p | 0.092 | 2.0e-03 | * |
| miR-362-5p | 0.086 | 2.1e-03 | * |
| miR-7160-5p | 0.088 | 2.2e-03 | * |
| miR-4465 | -0.126 | 2.2e-03 | * |
| miR-6075 | -0.072 | 2.2e-03 | * |
| miR-185-5p | 0.087 | 2.3e-03 | * |
| miR-4762-3p | -0.060 | 2.3e-03 | * |
| miR-2392 | -0.067 | 2.3e-03 | * |
| miR-6837-5p | 0.062 | 2.3e-03 | * |
| miR-4698 | 0.062 | 2.3e-03 | * |
| miR-371b-5p | -0.099 | 2.4e-03 | * |
| miR-4524b-3p | -0.048 | 2.4e-03 | * |
| miR-4485-5p | 0.144 | 2.4e-03 | * |
| miR-2909 | 0.068 | 2.5e-03 | * |
| miR-423-3p | 0.107 | 2.5e-03 | * |
| miR-29b-2-5p | -0.106 | 2.5e-03 | * |
| miR-4701-3p | -0.099 | 2.5e-03 | * |
| miR-4310 | 0.065 | 2.5e-03 | * |
| miR-1275 | -0.097 | 2.5e-03 | * |
| miR-3162-3p | 0.081 | 2.6e-03 | * |
| miR-596 | 0.070 | 2.7e-03 | * |
| miR-194-5p | 0.145 | 2.7e-03 | * |
| miR-520a-5p | 0.058 | 2.8e-03 | * |
| miR-7151-3p | -0.052 | 2.8e-03 | * |
| miR-376c-3p | -0.180 | 2.8e-03 | * |
| miR-181c-5p | -0.114 | 2.8e-03 | * |
| miR-424-5p | -0.233 | 2.8e-03 | * |
| miR-17-3p | -0.193 | 2.9e-03 | * |
| miR-532-3p | 0.120 | 2.9e-03 | * |
| miR-30e-3p | 0.128 | 3.0e-03 | * |
| miR-410-3p | -0.137 | 3.0e-03 | * |
| miR-6791-5p | -0.080 | 3.0e-03 | * |
| miR-892c-5p | 0.082 | 3.1e-03 | * |
| miR-191-3p | 0.071 | 3.2e-03 | * |
| miR-6790-5p | -0.053 | 3.2e-03 | * |
| miR-548f-3p | 0.057 | 3.2e-03 | * |
| miR-1304-3p | 0.074 | 3.2e-03 | * |
| miR-6818-5p | -0.056 | 3.4e-03 | * |
| miR-922 | 0.147 | 3.4e-03 | * |
| miR-4691-5p | 0.047 | 3.5e-03 | * |
| miR-548c-3p | 0.047 | 3.6e-03 | * |
| miR-3616-5p | 0.061 | 3.6e-03 | * |
| miR-1229-3p | 0.065 | 3.6e-03 | * |
| miR-519b-3p | -0.048 | 3.7e-03 | * |
| miR-874-5p | -0.052 | 3.7e-03 | * |
| miR-301b-3p | -0.092 | 3.8e-03 | * |
| miR-6797-3p | 0.062 | 3.8e-03 | * |
| miR-3940-5p | -0.082 | 3.8e-03 | * |
| miR-6866-5p | -0.052 | 3.8e-03 | * |
| miR-1306-3p | -0.071 | 3.9e-03 | * |
| miR-1228-3p | 0.068 | 4.0e-03 | * |
| miR-1199-5p | 0.104 | 4.0e-03 | * |
| miR-6775-3p | 0.061 | 4.0e-03 | * |
| miR-1238-3p | 0.072 | 4.1e-03 | * |
| miR-182-3p | -0.061 | 4.1e-03 | * |
| miR-4663 | 0.062 | 4.3e-03 | * |
| miR-4319 | 0.052 | 4.3e-03 | * |
| miR-6813-5p | -0.051 | 4.4e-03 | * |
| miR-3164 | 0.051 | 4.5e-03 | * |
| miR-6824-3p | 0.052 | 4.5e-03 | * |
| miR-3653-5p | -0.047 | 4.6e-03 | * |
| miR-4714-3p | -0.046 | 4.6e-03 | * |
| miR-4690-5p | -0.097 | 4.7e-03 | * |
| miR-449a | -0.071 | 4.7e-03 | * |
| miR-7852-3p | -0.059 | 4.8e-03 | * |

**Supplementary Table 8.** Comparison *HCvPSP*: Log fold changes and *p*-values for progressive supranuclear palsy (PSP, n=35) vs. controls (HC, n=416). No effect implies fold change = 1 and log fold change = 0. Significantly upregulated/downregulated miRNAs are highlighted in red/blue respectively. One asterisk means significant at an FDR of 5% (Benjamini-Hochberg), and three asterisks mean significant at an FWER of 5% (Bonferroni) across all comparisons.

| miRNA  upregulated n=126 downregulated n=117 | Effect size | p-value | Significance |
| --- | --- | --- | --- |
| miR-2115-5p | 0.491 | 1.3e-10 | *** |
| miR-1225-5p | 0.280 | 1.0e-06 | *** |
| miR-4762-3p | -0.225 | 4.1e-06 | *** |
| miR-4270 | 0.317 | 4.3e-06 | *** |
| miR-769-5p | 0.325 | 5.4e-06 | *** |
| miR-505-3p | 0.388 | 5.6e-06 | *** |
| miR-186-5p | 0.449 | 2.5e-05 | * |
| miR-940 | 0.270 | 2.7e-05 | * |
| miR-29c-5p | 0.431 | 3.5e-05 | * |
| miR-197-3p | 0.410 | 3.7e-05 | * |
| miR-4638-5p | 0.172 | 4.8e-05 | * |
| miR-4328 | 0.144 | 6.4e-05 | * |
| miR-92a-3p | 0.239 | 6.5e-05 | * |
| miR-7975 | -0.318 | 6.6e-05 | * |
| miR-151a-3p | 0.435 | 6.8e-05 | * |
| miR-501-5p | 0.288 | 8.9e-05 | * |
| miR-491-5p | 0.230 | 8.9e-05 | * |
| miR-361-5p | 0.317 | 1.1e-04 | * |
| miR-4465 | -0.354 | 2.1e-04 | * |
| miR-145-5p | 0.366 | 2.2e-04 | * |
| miR-6088 | 0.191 | 2.3e-04 | * |
| miR-130a-3p | -0.316 | 2.3e-04 | * |
| let-7d-3p | 0.319 | 2.4e-04 | * |
| miR-22-3p | 0.262 | 2.5e-04 | * |
| miR-1233-5p | -0.357 | 2.9e-04 | * |
| miR-6503-3p | 0.197 | 3.1e-04 | * |
| miR-6126 | -0.272 | 3.3e-04 | * |
| miR-6073 | -0.575 | 3.4e-04 | * |
| miR-4516 | 0.354 | 3.7e-04 | * |
| miR-6085 | -0.422 | 3.7e-04 | * |
| miR-30d-5p | 0.489 | 3.7e-04 | * |
| miR-378a-5p | 0.410 | 4.3e-04 | * |
| miR-4486 | -0.323 | 4.3e-04 | * |
| miR-1250-5p | 0.136 | 4.4e-04 | * |
| miR-345-5p | 0.176 | 4.6e-04 | * |
| miR-4291 | -0.325 | 5.2e-04 | * |
| miR-3614-5p | 0.166 | 5.3e-04 | * |
| miR-326 | 0.426 | 5.3e-04 | * |
| miR-4634 | 0.258 | 5.6e-04 | * |
| miR-5090 | -0.181 | 5.7e-04 | * |
| miR-125a-3p | -0.240 | 5.8e-04 | * |
| miR-23a-3p | 0.322 | 5.8e-04 | * |
| miR-502-3p | 0.247 | 7.1e-04 | * |
| miR-3923 | -0.162 | 7.4e-04 | * |
| miR-664b-3p | 0.234 | 7.7e-04 | * |
| miR-500a-3p | 0.222 | 8.2e-04 | * |
| miR-3653-5p | -0.133 | 8.4e-04 | * |
| miR-140-3p | 0.240 | 8.7e-04 | * |
| miR-373-3p | -0.134 | 8.7e-04 | * |
| miR-3065-3p | 0.134 | 9.8e-04 | * |
| miR-939-5p | -0.209 | 9.9e-04 | * |
| miR-5684 | -0.361 | 9.9e-04 | * |

**Supplementary Table 9.** Comparison HC_sex: Log fold changes and *p*-values for effect of sex in controls (HC, female:male 182:234) on miRNA expression. No effect implies fold change = 1 and log fold change = 0. Significantly upregulated/downregulated miRNAs are highlighted in red/blue respectively. One asterisk means significant at an FDR of 5% (Benjamini-Hochberg), and three asterisks mean significant at an FWER of 5% (Bonferroni) across all comparisons.

| *miRNA*  *upregulated n=126 downregulated n=93* | Effect size | p-value | Significance |
| --- | --- | --- | --- |
| miR-193b-3p | -0.320 | 2.6e-13 | *** |
| miR-503-5p | 0.339 | 1.8e-12 | *** |
| miR-1255b-5p | 0.256 | 3.9e-12 | *** |
| miR-6073 | 0.600 | 8.2e-12 | *** |
| miR-133b | -0.628 | 1.0e-10 | *** |
| miR-505-5p | 0.254 | 1.4e-09 | *** |
| miR-145-5p | -0.330 | 1.8e-09 | *** |
| miR-664a-5p | 0.282 | 3.3e-09 | *** |
| miR-103a-3p | 0.224 | 3.8e-09 | *** |
| miR-150-5p | -0.369 | 4.3e-09 | *** |
| miR-200c-3p | 0.183 | 1.3e-08 | *** |
| miR-223-3p | -0.236 | 1.7e-08 | *** |
| miR-106b-5p | 0.268 | 2.9e-08 | *** |
| miR-4753-5p | 0.136 | 4.5e-08 | *** |
| miR-4323 | -0.239 | 4.9e-08 | *** |
| miR-758-3p | -0.261 | 6.1e-08 | *** |
| miR-4737 | 0.401 | 6.8e-08 | *** |
| miR-590-5p | 0.614 | 9.5e-08 | *** |
| miR-34a-5p | 0.301 | 1.0e-07 | *** |
| miR-140-3p | -0.211 | 1.1e-07 | *** |
| miR-299-5p | -0.354 | 1.6e-07 | *** |
| miR-326 | -0.352 | 2.3e-07 | *** |
| miR-107 | 0.231 | 2.4e-07 | *** |
| miR-409-5p | -0.242 | 2.9e-07 | *** |
| miR-542-5p | 0.287 | 4.2e-07 | *** |
| miR-766-3p | -0.250 | 4.9e-07 | *** |
| miR-1273h-5p | 0.162 | 4.9e-07 | *** |
| miR-18b-5p | 0.498 | 5.1e-07 | *** |
| miR-29b-3p | 0.330 | 6.4e-07 | *** |
| miR-133a-3p | -0.163 | 6.5e-07 | *** |
| miR-654-3p | -0.490 | 6.8e-07 | *** |
| miR-23b-3p | -0.241 | 6.9e-07 | *** |
| miR-769-3p | 0.104 | 7.1e-07 | *** |
| miR-936 | 0.178 | 7.1e-07 | *** |
| miR-122-5p | 0.276 | 1.0e-06 | *** |
| miR-130b-3p | 0.164 | 1.1e-06 | *** |
| miR-6088 | -0.138 | 1.1e-06 | *** |
| miR-338-5p | 0.154 | 1.3e-06 | *** |
| miR-892b | 0.237 | 1.3e-06 | *** |
| miR-579-5p | 0.103 | 1.4e-06 | *** |
| miR-3179 | 0.168 | 1.7e-06 | *** |
| miR-409-3p | -0.448 | 2.0e-06 | *** |
| miR-130a-3p | 0.221 | 2.2e-06 | *** |
| miR-4443 | 0.281 | 2.5e-06 | *** |
| miR-30a-3p | -0.178 | 2.9e-06 | *** |
| miR-181c-3p | -0.219 | 3.0e-06 | *** |
| miR-1912 | -0.156 | 3.2e-06 | *** |
| let-7c-5p | 0.315 | 3.3e-06 | *** |
| miR-1185-1-3p | -0.180 | 3.6e-06 | *** |
| miR-411-3p | -0.126 | 3.8e-06 | *** |
| miR-7975 | 0.202 | 3.9e-06 | *** |
| miR-34b-5p | 0.122 | 4.0e-06 | *** |
| miR-3127-5p | 0.219 | 4.4e-06 | *** |
| miR-6780a-5p | 0.239 | 4.9e-06 | *** |
| miR-29b-2-5p | 0.223 | 5.3e-06 | *** |
| miR-3652 | 0.227 | 5.5e-06 | *** |
| miR-4742-5p | 0.246 | 5.5e-06 | *** |
| miR-1225-5p | -0.138 | 6.6e-06 | *** |
| miR-1271-5p | -0.204 | 7.2e-06 | *** |
| miR-23a-3p | -0.232 | 7.6e-06 | *** |
| miR-485-3p | -0.414 | 8.9e-06 | *** |
| miR-132-3p | 0.174 | 8.9e-06 | *** |
| miR-199a-5p | -0.342 | 9.4e-06 | *** |
| miR-342-3p | -0.209 | 9.6e-06 | *** |
| miR-18a-5p | 0.474 | 1.0e-05 | *** |
| miR-3907 | 0.321 | 1.0e-05 | *** |
| miR-1255a | 0.152 | 1.0e-05 | *** |
| miR-505-3p | -0.209 | 1.0e-05 | *** |
| miR-6134 | 0.141 | 1.2e-05 | *** |
| miR-6792-5p | 0.145 | 1.2e-05 | *** |
| miR-154-5p | -0.331 | 1.3e-05 | *** |
| miR-361-5p | -0.196 | 1.4e-05 | *** |
| miR-148a-3p | 0.329 | 1.5e-05 | *** |
| miR-3156-5p | 0.167 | 1.5e-05 | *** |
| miR-548aw | 0.115 | 1.6e-05 | *** |
| miR-134-3p | -0.103 | 1.8e-05 | *** |
| miR-543 | -0.277 | 1.8e-05 | *** |
| miR-5581-5p | 0.212 | 2.1e-05 | * |
| miR-329-3p | -0.280 | 2.1e-05 | * |
| miR-127-3p | -0.252 | 2.3e-05 | * |
| miR-369-5p | -0.127 | 2.4e-05 | * |
| miR-495-3p | -0.347 | 2.6e-05 | * |
| miR-4281 | -0.161 | 2.6e-05 | * |
| miR-6767-5p | 0.186 | 2.6e-05 | * |
| miR-377-3p | -0.319 | 2.7e-05 | * |
| miR-3163 | 0.181 | 2.7e-05 | * |
| miR-1288-3p | 0.191 | 2.9e-05 | * |
| miR-323a-3p | -0.301 | 2.9e-05 | * |
| miR-6754-5p | 0.100 | 3.7e-05 | * |
| miR-5088-5p | 0.202 | 4.2e-05 | * |
| miR-31-5p | -0.214 | 4.5e-05 | * |
| miR-1295a | 0.133 | 4.5e-05 | * |
| miR-208a-5p | 0.117 | 5.4e-05 | * |
| miR-1275 | -0.165 | 5.5e-05 | * |
| miR-4685-5p | 0.293 | 6.2e-05 | * |
| miR-6734-5p | 0.191 | 6.4e-05 | * |
| miR-5194 | 0.167 | 7.1e-05 | * |
| miR-1305 | 0.183 | 7.5e-05 | * |
| miR-3691-5p | 0.088 | 7.7e-05 | * |
| miR-629-3p | -0.186 | 7.9e-05 | * |
| miR-496 | -0.101 | 8.4e-05 | * |
| miR-4716-3p | 0.206 | 8.4e-05 | * |
| miR-6856-5p | 0.125 | 9.0e-05 | * |
| let-7i-5p | 0.215 | 9.2e-05 | * |
| miR-6821-5p | -0.121 | 1.1e-04 | * |
| miR-6512-5p | 0.195 | 1.2e-04 | * |
| miR-3125 | 0.196 | 1.2e-04 | * |
| miR-940 | -0.136 | 1.2e-04 | * |
| miR-610 | 0.148 | 1.3e-04 | * |
| miR-410-3p | -0.238 | 1.3e-04 | * |
| miR-493-5p | -0.195 | 1.3e-04 | * |
| miR-3189-5p | 0.104 | 1.4e-04 | * |
| miR-378g | 0.236 | 1.4e-04 | * |
| miR-99b-5p | -0.225 | 1.5e-04 | * |
| miR-1304-5p | 0.097 | 1.6e-04 | * |
| miR-1185-2-3p | -0.111 | 1.6e-04 | * |
| miR-7-5p | 0.242 | 1.8e-04 | * |
| miR-4516 | -0.206 | 1.8e-04 | * |
| miR-6807-5p | 0.166 | 1.8e-04 | * |
| miR-642b-5p | -0.100 | 1.9e-04 | * |
| miR-4659b-3p | -0.192 | 1.9e-04 | * |
| miR-4514 | 0.116 | 2.0e-04 | * |
| miR-4784 | 0.116 | 2.6e-04 | * |
| let-7b-5p | 0.263 | 2.7e-04 | * |
| miR-4513 | 0.229 | 2.8e-04 | * |
| miR-6720-3p | 0.115 | 2.9e-04 | * |
| miR-6090 | -0.142 | 2.9e-04 | * |
| miR-139-5p | -0.195 | 3.1e-04 | * |
| miR-21-3p | 0.261 | 3.1e-04 | * |
| miR-3176 | 0.153 | 3.3e-04 | * |
| miR-376a-3p | -0.334 | 3.3e-04 | * |
| miR-3120-3p | -0.110 | 3.5e-04 | * |
| miR-4659b-5p | -0.134 | 3.7e-04 | * |
| miR-6794-5p | 0.138 | 3.7e-04 | * |
| miR-6131 | 0.173 | 4.2e-04 | * |
| miR-3135b | 0.259 | 4.2e-04 | * |
| miR-4713-3p | 0.174 | 4.4e-04 | * |
| miR-5003-5p | 0.125 | 4.6e-04 | * |
| miR-337-3p | -0.287 | 4.7e-04 | * |
| miR-4653-3p | 0.175 | 4.9e-04 | * |
| miR-4779 | 0.105 | 5.4e-04 | * |
| miR-3140-3p | 0.107 | 5.5e-04 | * |
| miR-3161 | 0.102 | 5.7e-04 | * |
| miR-381-3p | -0.188 | 5.9e-04 | * |
| miR-614 | 0.089 | 6.3e-04 | * |
| miR-376a-5p | -0.189 | 6.3e-04 | * |
| miR-424-5p | 0.352 | 6.8e-04 | * |
| miR-101-3p | 0.394 | 7.0e-04 | * |
| miR-3190-3p | 0.087 | 7.1e-04 | * |
| miR-3198 | 0.170 | 7.2e-04 | * |
| miR-433-3p | -0.149 | 7.2e-04 | * |
| miR-3960 | -0.170 | 8.1e-04 | * |
| miR-1914-3p | 0.158 | 9.2e-04 | * |
| miR-7152-3p | 0.153 | 9.9e-04 | * |
| miR-4289 | 0.096 | 9.9e-04 | * |
| miR-340-5p | 0.295 | 1.0e-03 | * |
| miR-6717-5p | 0.149 | 1.0e-03 | * |
| miR-93-3p | -0.146 | 1.0e-03 | * |
| miR-1226-5p | 0.087 | 1.0e-03 | * |
| miR-29a-3p | -0.171 | 1.1e-03 | * |
| miR-330-3p | -0.128 | 1.1e-03 | * |
| miR-335-3p | -0.121 | 1.1e-03 | * |
| miR-6510-5p | 0.178 | 1.2e-03 | * |
| miR-497-5p | 0.120 | 1.2e-03 | * |
| miR-423-3p | -0.144 | 1.3e-03 | * |
| miR-26a-1-3p | -0.065 | 1.3e-03 | * |
| miR-654-5p | -0.093 | 1.3e-03 | * |
| miR-4657 | 0.086 | 1.3e-03 | * |
| miR-202-3p | 0.089 | 1.3e-03 | * |
| miR-660-5p | 0.216 | 1.3e-03 | * |
| miR-378f | 0.152 | 1.4e-03 | * |
| miR-487b-3p | -0.224 | 1.4e-03 | * |
| miR-101-5p | 0.265 | 1.4e-03 | * |
| miR-129-1-3p | -0.108 | 1.5e-03 | * |
| miR-4442 | 0.145 | 1.5e-03 | * |
| miR-1233-5p | 0.176 | 1.6e-03 | * |
| miR-5193 | -0.065 | 1.6e-03 | * |
| miR-668-3p | -0.086 | 1.6e-03 | * |
| miR-4648 | 0.087 | 1.6e-03 | * |
| miR-6829-5p | 0.092 | 1.6e-03 | * |
| miR-579-3p | 0.096 | 1.7e-03 | * |
| miR-193a-3p | 0.205 | 1.7e-03 | * |
| miR-4659a-3p | -0.149 | 1.7e-03 | * |
| miR-513c-3p | 0.060 | 1.8e-03 | * |
| miR-1307-5p | 0.102 | 1.8e-03 | * |
| miR-10a-5p | -0.203 | 2.0e-03 | * |
| miR-22-5p | 0.171 | 2.1e-03 | * |
| miR-10b-5p | -0.086 | 2.1e-03 | * |
| miR-6757-5p | 0.155 | 2.2e-03 | * |
| miR-4518 | 0.110 | 2.2e-03 | * |
| miR-136-5p | -0.118 | 2.2e-03 | * |
| let-7e-5p | 0.221 | 2.2e-03 | * |
| miR-5701 | 0.097 | 2.3e-03 | * |
| miR-4466 | -0.106 | 2.3e-03 | * |
| miR-6879-5p | 0.150 | 2.5e-03 | * |
| miR-5581-3p | -0.068 | 2.7e-03 | * |
| miR-1236-5p | 0.109 | 2.7e-03 | * |
| miR-224-5p | -0.170 | 2.8e-03 | * |
| miR-6130 | -0.115 | 2.9e-03 | * |
| miR-6800-5p | -0.111 | 3.0e-03 | * |
| miR-491-5p | -0.096 | 3.1e-03 | * |
| let-7d-5p | 0.148 | 3.3e-03 | * |
| miR-146b-5p | -0.238 | 3.3e-03 | * |
| miR-181a-2-3p | -0.119 | 3.4e-03 | * |
| miR-663a | -0.115 | 3.5e-03 | * |
| miR-6085 | 0.191 | 3.5e-03 | * |
| miR-4268 | -0.072 | 3.6e-03 | * |
| miR-3936 | 0.076 | 3.6e-03 | * |
| miR-3165 | -0.070 | 3.8e-03 | * |
| miR-4465 | 0.156 | 3.8e-03 | * |
| miR-212-3p | 0.138 | 3.9e-03 | * |
| miR-5591-3p | -0.058 | 3.9e-03 | * |
| miR-3607-3p | 0.091 | 4.0e-03 | * |
| miR-363-5p | 0.080 | 4.0e-03 | * |
| miR-378d | 0.153 | 4.0e-03 | * |
| miR-4470 | 0.078 | 4.1e-03 | * |
| miR-608 | -0.072 | 4.1e-03 | * |
| miR-431-5p | -0.147 | 4.2e-03 | * |
| miR-3665 | -0.105 | 4.3e-03 | * |

**Supplementary Table 10.** Comparison *PD_sex*: Log fold changes and *p*-values for effect of sex in Parkinson’s disease (PD, female:male 116:251) on miRNA expression. No effect implies fold change = 1 and log fold change = 0. Significantly upregulated/downregulated miRNAs are highlighted in red/blue respectively. One asterisk means significant at an FDR of 5% (Benjamini-Hochberg), and three asterisks mean significant at an FWER of 5% (Bonferroni) across all comparisons.

| *miRNA*  *upregulated n=12 downregulated n=32* | Effect size | p-value | Significance |
| --- | --- | --- | --- |
| miR-3940-5p | -0.211 | 3.7e-06 | *** |
| miR-224-5p | -0.291 | 7.2e-06 | *** |
| miR-193b-3p | -0.223 | 1.1e-05 | *** |
| miR-6789-5p | -0.354 | 1.8e-05 | *** |
| miR-150-5p | -0.299 | 2.0e-05 | * |
| miR-133b | -0.495 | 2.1e-05 | * |
| miR-133a-3p | -0.175 | 2.4e-05 | * |
| miR-130b-3p | 0.184 | 2.7e-05 | * |
| miR-1249-5p | -0.188 | 2.8e-05 | * |
| miR-503-5p | 0.243 | 3.4e-05 | * |
| miR-7-5p | 0.375 | 3.9e-05 | * |
| miR-6779-5p | -0.238 | 5.0e-05 | * |
| miR-3616-3p | -0.195 | 6.7e-05 | * |
| miR-31-5p | -0.225 | 7.4e-05 | * |
| miR-136-3p | -0.138 | 7.9e-05 | * |
| miR-625-5p | -0.210 | 8.2e-05 | * |
| miR-1228-5p | 0.216 | 9.0e-05 | * |
| miR-3665 | -0.176 | 9.5e-05 | * |
| miR-3196 | -0.179 | 9.5e-05 | * |
| miR-4443 | 0.258 | 1.3e-04 | * |
| miR-664a-5p | 0.246 | 1.3e-04 | * |
| miR-4532 | -0.114 | 1.4e-04 | * |
| miR-34a-5p | 0.253 | 1.4e-04 | * |
| miR-1912 | -0.135 | 1.8e-04 | * |
| miR-129-1-3p | -0.122 | 2.0e-04 | * |
| miR-3656 | -0.263 | 2.4e-04 | * |
| miR-4742-5p | 0.224 | 2.4e-04 | * |
| miR-744-5p | -0.179 | 2.5e-04 | * |
| miR-6803-5p | -0.314 | 2.9e-04 | * |
| miR-6850-5p | -0.158 | 3.2e-04 | * |
| miR-4665-5p | -0.254 | 3.5e-04 | * |
| miR-664b-5p | 0.400 | 4.0e-04 | * |
| miR-424-3p | 0.198 | 4.1e-04 | * |
| miR-6852-5p | -0.118 | 4.2e-04 | * |
| miR-3188 | -0.105 | 4.5e-04 | * |
| miR-6786-5p | -0.149 | 4.5e-04 | * |
| miR-125b-1-3p | -0.182 | 4.6e-04 | * |
| miR-4466 | -0.156 | 4.7e-04 | * |
| miR-6871-5p | 0.160 | 5.1e-04 | * |
| miR-1911-3p | 0.085 | 5.3e-04 | * |
| miR-625-3p | -0.158 | 5.8e-04 | * |
| miR-4672 | -0.151 | 8.1e-04 | * |
| miR-802 | -0.117 | 8.4e-04 | * |
| miR-4323 | -0.187 | 8.6e-04 | * |

**Supplementary Table 11.** Comparison PSP_sex: Log fold changes and *p*-values for effect of sex in progressive supranuclear palsy (PSP, female:male 15:20) on miRNA expression. No effect implies fold change = 1 and log fold change = 0. Significantly upregulated/downregulated miRNAs are highlighted in red/blue respectively. One asterisk means significant at an FDR of 5% (Benjamini-Hochberg), and three asterisks mean significant at an FWER of 5% (Bonferroni) across all comparisons.

| *miRNA*  *upregulated n=0 downregulated n=2* | Effect size | p-value | Significance |
| --- | --- | --- | --- |
| miR-3688-5p | -0.356 | 1.0e-05 | *** |
| miR-511-3p | -0.489 | 1.6e-05 | *** |

**Supplementary Table 12.** Comparison *HC_AAA*: Log fold changes and *p*-values for effect of age at assessment (AAA) in controls (HC, n=416) on miRNA expression. No effect implies fold change = 1 and log fold change = 0. Significantly upregulated/downregulated miRNAs are highlighted in red/blue respectively. One asterisk means significant at an FDR of 5% (Benjamini-Hochberg), and three asterisks mean significant at an FWER of 5% (Bonferroni) across all comparisons.

| *miRNA*  *upregulated n=98 downregulated n=106* | Effect size | p-value | Significance |
| --- | --- | --- | --- |
| miR-31-5p | -0.015 | 5.4e-12 | *** |
| miR-196b-5p | -0.021 | 1.8e-10 | *** |
| miR-199a-3p | -0.032 | 5.6e-10 | *** |
| miR-20a-3p | -0.021 | 2.4e-09 | *** |
| miR-20a-5p | -0.022 | 1.6e-08 | *** |
| miR-598-3p | -0.015 | 1.7e-08 | *** |
| miR-29c-5p | 0.014 | 2.3e-08 | *** |
| miR-17-5p | -0.015 | 3.1e-08 | *** |
| miR-454-3p | -0.026 | 4.4e-08 | *** |
| miR-27b-3p | -0.017 | 1.6e-07 | *** |
| miR-4516 | 0.012 | 3.3e-07 | *** |
| miR-16-5p | -0.012 | 7.6e-07 | *** |
| miR-126-3p | -0.024 | 8.1e-07 | *** |
| miR-15a-5p | -0.017 | 1.2e-06 | *** |
| miR-17-3p | -0.018 | 1.2e-06 | *** |
| miR-432-5p | -0.008 | 1.3e-06 | *** |
| miR-1973 | 0.011 | 1.5e-06 | *** |
| miR-126-5p | -0.026 | 1.5e-06 | *** |
| miR-144-5p | -0.034 | 1.5e-06 | *** |
| miR-548am-5p | -0.006 | 1.5e-06 | *** |
| miR-379-5p | -0.008 | 2.5e-06 | *** |
| miR-146b-5p | -0.016 | 2.5e-06 | *** |
| miR-539-5p | -0.006 | 3.1e-06 | *** |
| miR-20b-5p | -0.017 | 3.2e-06 | *** |
| miR-487b-3p | -0.014 | 3.5e-06 | *** |
| miR-15b-5p | -0.008 | 3.7e-06 | *** |
| miR-18a-5p | -0.021 | 4.3e-06 | *** |
| miR-6821-5p | 0.006 | 5.9e-06 | *** |
| miR-548d-5p | -0.005 | 7.0e-06 | *** |
| miR-643 | -0.006 | 7.3e-06 | *** |
| miR-95-3p | -0.005 | 7.9e-06 | *** |
| miR-1207-5p | 0.006 | 8.4e-06 | *** |
| miR-197-5p | 0.008 | 9.0e-06 | *** |
| miR-494-3p | 0.015 | 9.3e-06 | *** |
| miR-195-5p | -0.021 | 9.9e-06 | *** |
| miR-493-5p | -0.010 | 1.0e-05 | *** |
| miR-642a-3p | 0.008 | 1.0e-05 | *** |
| miR-337-5p | -0.008 | 1.2e-05 | *** |
| miR-6090 | 0.007 | 1.2e-05 | *** |
| miR-144-3p | -0.032 | 1.3e-05 | *** |
| miR-101-3p | -0.021 | 1.4e-05 | *** |
| miR-4515 | 0.005 | 1.4e-05 | *** |
| miR-140-5p | -0.015 | 1.6e-05 | *** |
| miR-4284 | 0.013 | 1.7e-05 | *** |
| miR-374a-5p | -0.026 | 2.0e-05 | * |
| miR-299-5p | -0.012 | 2.1e-05 | * |
| miR-301a-3p | -0.023 | 2.1e-05 | * |
| miR-624-5p | -0.016 | 2.5e-05 | * |
| miR-3162-5p | 0.006 | 2.6e-05 | * |
| let-7g-5p | -0.016 | 2.7e-05 | * |
| miR-26b-5p | -0.020 | 3.1e-05 | * |
| miR-32-5p | -0.021 | 3.2e-05 | * |
| miR-664b-5p | 0.015 | 3.5e-05 | * |
| miR-98-5p | -0.017 | 3.7e-05 | * |
| miR-369-5p | -0.005 | 3.8e-05 | * |
| miR-7641 | 0.011 | 3.9e-05 | * |
| miR-190a-5p | -0.017 | 4.2e-05 | * |
| miR-1291 | 0.007 | 4.2e-05 | * |
| miR-1225-5p | 0.005 | 4.9e-05 | * |
| miR-221-5p | -0.009 | 5.1e-05 | * |
| miR-579-3p | -0.005 | 5.3e-05 | * |
| miR-550b-2-5p | -0.008 | 5.3e-05 | * |
| miR-4485-3p | 0.009 | 5.3e-05 | * |
| miR-96-5p | -0.018 | 5.7e-05 | * |
| miR-374b-5p | -0.017 | 5.8e-05 | * |
| miR-18b-5p | -0.017 | 6.0e-05 | * |
| miR-424-5p | -0.018 | 6.4e-05 | * |
| miR-92a-3p | 0.006 | 7.5e-05 | * |
| miR-1202 | 0.005 | 8.0e-05 | * |
| miR-3960 | 0.009 | 8.1e-05 | * |
| miR-30d-5p | 0.013 | 8.9e-05 | * |
| miR-376c-3p | -0.013 | 9.0e-05 | * |
| miR-22-3p | 0.007 | 9.1e-05 | * |
| miR-1181 | 0.005 | 9.2e-05 | * |
| miR-301b-3p | -0.007 | 1.1e-04 | * |
| miR-4485-5p | 0.010 | 1.2e-04 | * |
| miR-93-5p | -0.009 | 1.3e-04 | * |
| miR-6132 | 0.011 | 1.3e-04 | * |
| miR-325 | -0.004 | 1.4e-04 | * |
| miR-431-5p | -0.008 | 1.4e-04 | * |
| miR-6749-5p | 0.007 | 1.5e-04 | * |
| miR-200b-3p | -0.005 | 1.5e-04 | * |
| miR-4289 | -0.005 | 1.5e-04 | * |
| miR-495-3p | -0.013 | 1.9e-04 | * |
| miR-4318 | -0.009 | 2.0e-04 | * |
| miR-3195 | 0.006 | 2.0e-04 | * |
| miR-410-3p | -0.010 | 2.2e-04 | * |
| let-7f-5p | -0.010 | 2.3e-04 | * |
| miR-192-3p | -0.009 | 2.3e-04 | * |
| miR-4281 | 0.006 | 2.6e-04 | * |
| miR-1290 | 0.006 | 2.7e-04 | * |
| miR-3136-5p | -0.004 | 2.8e-04 | * |
| miR-4651 | 0.004 | 2.9e-04 | * |
| let-7i-3p | -0.004 | 3.0e-04 | * |
| miR-3674 | 0.004 | 3.1e-04 | * |
| miR-654-3p | -0.015 | 3.2e-04 | * |
| miR-762 | 0.005 | 3.3e-04 | * |
| miR-221-3p | -0.008 | 3.6e-04 | * |
| miR-21-5p | -0.010 | 3.8e-04 | * |
| miR-8063 | 0.007 | 3.8e-04 | * |
| miR-4739 | 0.005 | 3.8e-04 | * |
| miR-6752-5p | 0.004 | 3.8e-04 | * |
| miR-34a-5p | 0.009 | 3.8e-04 | * |
| miR-6127 | 0.009 | 3.9e-04 | * |
| miR-7846-3p | 0.010 | 3.9e-04 | * |
| miR-150-3p | 0.005 | 4.1e-04 | * |
| miR-6087 | 0.007 | 4.2e-04 | * |
| miR-4721 | 0.008 | 4.4e-04 | * |
| miR-4728-5p | 0.009 | 4.4e-04 | * |
| miR-873-5p | 0.004 | 4.6e-04 | * |
| miR-1246 | 0.014 | 5.0e-04 | * |
| miR-19b-1-5p | -0.005 | 5.5e-04 | * |
| let-7a-5p | -0.008 | 5.7e-04 | * |
| miR-6875-5p | 0.008 | 5.9e-04 | * |
| miR-1273g-3p | 0.010 | 6.7e-04 | * |
| miR-620 | -0.006 | 6.8e-04 | * |
| miR-106b-3p | -0.004 | 6.9e-04 | * |
| miR-8069 | 0.008 | 7.2e-04 | * |
| miR-6850-5p | 0.005 | 7.4e-04 | * |
| miR-377-3p | -0.011 | 8.0e-04 | * |
| miR-551b-3p | -0.004 | 8.2e-04 | * |
| miR-4327 | 0.004 | 8.9e-04 | * |
| miR-6880-5p | 0.005 | 9.1e-04 | * |
| miR-2276-3p | 0.003 | 9.2e-04 | * |
| miR-337-3p | -0.012 | 9.5e-04 | * |
| miR-193b-3p | -0.006 | 9.6e-04 | * |
| miR-4634 | 0.006 | 9.6e-04 | * |
| miR-4532 | 0.004 | 9.8e-04 | * |
| miR-662 | 0.003 | 9.8e-04 | * |
| miR-3651 | 0.007 | 1.0e-03 | * |
| miR-542-3p | -0.005 | 1.1e-03 | * |
| miR-6728-5p | 0.005 | 1.1e-03 | * |
| miR-425-5p | 0.006 | 1.1e-03 | * |
| miR-6826-5p | 0.007 | 1.1e-03 | * |
| miR-3137 | 0.005 | 1.2e-03 | * |
| miR-4763-3p | 0.005 | 1.2e-03 | * |
| miR-509-3-5p | 0.003 | 1.2e-03 | * |
| miR-921 | 0.004 | 1.2e-03 | * |
| miR-5006-5p | 0.006 | 1.2e-03 | * |
| miR-7-5p | -0.009 | 1.3e-03 | * |
| let-7d-5p | -0.007 | 1.3e-03 | * |
| miR-25-3p | 0.004 | 1.3e-03 | * |
| miR-4428 | 0.007 | 1.3e-03 | * |
| miR-30b-3p | -0.003 | 1.3e-03 | * |
| miR-148a-3p | -0.010 | 1.4e-03 | * |
| miR-6818-5p | -0.004 | 1.4e-03 | * |
| miR-3163 | -0.006 | 1.4e-03 | * |
| miR-329-3p | -0.009 | 1.4e-03 | * |
| miR-6865-5p | 0.004 | 1.4e-03 | * |
| miR-4306 | 0.005 | 1.5e-03 | * |
| miR-3663-3p | 0.005 | 1.5e-03 | * |
| miR-1827 | -0.006 | 1.5e-03 | * |
| miR-141-3p | -0.005 | 1.5e-03 | * |
| miR-4999-5p | -0.004 | 1.5e-03 | * |
| miR-3120-3p | -0.004 | 1.6e-03 | * |
| miR-4697-5p | 0.013 | 1.6e-03 | * |
| miR-361-5p | 0.006 | 1.7e-03 | * |
| miR-6894-5p | 0.004 | 1.7e-03 | * |
| miR-758-3p | -0.007 | 1.7e-03 | * |
| miR-6738-5p | 0.004 | 1.8e-03 | * |
| miR-6872-5p | 0.004 | 1.8e-03 | * |
| miR-450a-5p | -0.005 | 1.8e-03 | * |
| miR-6780b-5p | 0.006 | 1.8e-03 | * |
| let-7i-5p | -0.007 | 1.8e-03 | * |
| miR-4655-3p | 0.011 | 2.0e-03 | * |
| miR-6800-5p | 0.005 | 2.0e-03 | * |
| miR-376a-3p | -0.012 | 2.0e-03 | * |
| miR-3654 | 0.004 | 2.1e-03 | * |
| miR-628-5p | -0.006 | 2.1e-03 | * |
| miR-365a-3p | 0.007 | 2.1e-03 | * |
| miR-4500 | -0.005 | 2.2e-03 | * |
| miR-374c-5p | -0.012 | 2.2e-03 | * |
| miR-4746-3p | 0.004 | 2.3e-03 | * |
| miR-3650 | -0.003 | 2.3e-03 | * |
| miR-324-3p | 0.006 | 2.3e-03 | * |
| miR-151a-3p | 0.008 | 2.4e-03 | * |
| miR-500a-5p | 0.005 | 2.4e-03 | * |
| miR-127-3p | -0.008 | 2.4e-03 | * |
| miR-4796-3p | -0.003 | 2.4e-03 | * |
| miR-4798-5p | -0.003 | 2.5e-03 | * |
| miR-3648 | 0.004 | 2.5e-03 | * |
| miR-513a-5p | 0.007 | 2.5e-03 | * |
| miR-582-5p | -0.009 | 2.6e-03 | * |
| miR-128-1-5p | 0.004 | 2.6e-03 | * |
| miR-6747-5p | 0.005 | 2.7e-03 | * |
| miR-660-5p | -0.009 | 2.7e-03 | * |
| miR-4656 | 0.005 | 2.7e-03 | * |
| miR-5581-3p | 0.003 | 2.8e-03 | * |
| miR-376a-5p | -0.007 | 2.8e-03 | * |
| miR-3909 | 0.006 | 2.9e-03 | * |
| miR-6806-5p | 0.004 | 3.0e-03 | * |
| miR-3923 | -0.003 | 3.1e-03 | * |
| miR-140-3p | 0.005 | 3.2e-03 | * |
| miR-1229-5p | 0.005 | 3.3e-03 | * |
| miR-543 | -0.008 | 3.3e-03 | * |
| let-7e-5p | -0.009 | 3.4e-03 | * |
| miR-212-5p | 0.002 | 3.4e-03 | * |
| miR-665 | 0.006 | 3.5e-03 | * |
| miR-5190 | 0.003 | 3.5e-03 | * |
| miR-576-3p | -0.004 | 3.6e-03 | * |
| miR-1250-3p | -0.003 | 3.7e-03 | * |
| miR-23a-3p | 0.006 | 3.8e-03 | * |
| miR-4743-5p | 0.004 | 3.8e-03 | * |
| miR-1226-5p | 0.003 | 3.9e-03 | * |

**Supplementary Table 13.** Comparison *PD_AAA*: Log fold changes and *p*-values for effect of age at assessment (AAA) in Parkinson’s disease (PD, n=367) on miRNA expression. No effect implies fold change = 1 and log fold change = 0. Significantly upregulated/downregulated miRNAs are highlighted in red/blue respectively. One asterisk means significant at an FDR of 5% (Benjamini-Hochberg), and three asterisks mean significant at an FWER of 5% (Bonferroni) across all comparisons.

| *miRNA*  *upregulated n=60 downregulated n=49* | Effect size | p-value | Significance |
| --- | --- | --- | --- |
| miR-665 | 0.012 | 1.2e-09 | *** |
| miR-22-3p | 0.011 | 4.1e-09 | *** |
| miR-31-5p | -0.013 | 1.5e-08 | *** |
| miR-20a-3p | -0.022 | 1.4e-07 | *** |
| let-7d-5p | -0.012 | 3.0e-07 | *** |
| miR-199a-3p | -0.029 | 4.2e-07 | *** |
| miR-196b-5p | -0.020 | 4.2e-07 | *** |
| miR-6073 | -0.022 | 8.0e-07 | *** |
| miR-93-5p | -0.015 | 8.4e-07 | *** |
| miR-25-3p | 0.008 | 9.3e-07 | *** |
| miR-20a-5p | -0.023 | 9.4e-07 | *** |
| let-7e-5p | -0.015 | 1.7e-06 | *** |
| miR-126-3p | -0.025 | 2.1e-06 | *** |
| miR-20b-5p | -0.020 | 2.6e-06 | *** |
| miR-454-3p | -0.026 | 4.3e-06 | *** |
| miR-29c-5p | 0.014 | 4.4e-06 | *** |
| miR-3135b | -0.019 | 6.3e-06 | *** |
| miR-30d-5p | 0.018 | 7.0e-06 | *** |
| miR-140-3p | 0.008 | 1.1e-05 | *** |
| miR-98-5p | -0.018 | 1.1e-05 | *** |
| miR-642a-3p | 0.008 | 1.1e-05 | *** |
| miR-361-5p | 0.010 | 1.6e-05 | *** |
| miR-126-5p | -0.024 | 1.6e-05 | *** |
| miR-17-5p | -0.015 | 1.9e-05 | *** |
| miR-34a-5p | 0.011 | 3.3e-05 | * |
| miR-1275 | -0.009 | 3.3e-05 | * |
| miR-550a-3p | 0.013 | 3.5e-05 | * |
| miR-2115-5p | 0.010 | 3.9e-05 | * |
| miR-29b-2-5p | -0.009 | 4.1e-05 | * |
| miR-378a-5p | 0.013 | 4.3e-05 | * |
| miR-584-3p | 0.008 | 4.5e-05 | * |
| miR-30a-5p | 0.017 | 4.6e-05 | * |
| miR-185-5p | 0.007 | 5.3e-05 | * |
| miR-144-5p | -0.032 | 6.2e-05 | * |
| miR-151a-3p | 0.012 | 6.3e-05 | * |
| miR-181c-5p | -0.011 | 6.9e-05 | * |
| miR-769-5p | 0.009 | 7.1e-05 | * |
| miR-3180-3p | 0.014 | 7.8e-05 | * |
| miR-221-3p | -0.011 | 7.9e-05 | * |
| miR-130a-3p | -0.011 | 8.1e-05 | * |
| miR-16-5p | -0.011 | 8.3e-05 | * |
| miR-2681-5p | 0.007 | 8.7e-05 | * |
| let-7a-5p | -0.010 | 9.3e-05 | * |
| miR-26b-5p | -0.020 | 9.9e-05 | * |
| miR-29a-3p | 0.011 | 1.0e-04 | * |
| miR-32-5p | -0.019 | 1.0e-04 | * |
| miR-659-3p | 0.006 | 1.0e-04 | * |
| miR-7641 | 0.012 | 1.1e-04 | * |
| miR-301a-3p | -0.025 | 1.1e-04 | * |
| miR-548q | -0.007 | 1.2e-04 | * |
| miR-582-3p | 0.007 | 1.3e-04 | * |
| miR-30c-5p | 0.014 | 1.6e-04 | * |
| miR-4270 | 0.008 | 1.6e-04 | * |
| miR-15b-5p | -0.006 | 1.9e-04 | * |
| miR-1225-5p | 0.006 | 2.1e-04 | * |
| let-7f-5p | -0.011 | 2.4e-04 | * |
| miR-502-3p | 0.008 | 2.5e-04 | * |
| miR-210-3p | 0.009 | 2.6e-04 | * |
| miR-4306 | 0.006 | 2.7e-04 | * |
| miR-505-3p | 0.009 | 3.1e-04 | * |
| miR-4788 | 0.013 | 3.6e-04 | * |
| miR-500a-3p | 0.006 | 3.6e-04 | * |
| miR-4500 | -0.006 | 3.7e-04 | * |
| miR-6749-5p | 0.009 | 3.7e-04 | * |
| miR-148a-5p | 0.006 | 3.9e-04 | * |
| miR-548d-5p | -0.005 | 4.0e-04 | * |
| miR-29b-1-5p | -0.009 | 4.2e-04 | * |
| miR-7847-3p | 0.008 | 4.5e-04 | * |
| miR-589-3p | 0.007 | 5.0e-04 | * |
| miR-7152-3p | 0.009 | 5.4e-04 | * |
| miR-27b-3p | -0.014 | 5.5e-04 | * |
| miR-221-5p | -0.008 | 5.8e-04 | * |
| miR-3610 | 0.006 | 5.9e-04 | * |
| miR-101-3p | -0.020 | 6.0e-04 | * |
| miR-659-5p | 0.005 | 6.0e-04 | * |
| miR-18a-3p | 0.010 | 6.1e-04 | * |
| miR-500a-5p | 0.007 | 6.2e-04 | * |
| miR-139-3p | -0.006 | 6.4e-04 | * |
| miR-18a-5p | -0.019 | 6.6e-04 | * |
| miR-493-5p | -0.008 | 6.9e-04 | * |
| miR-425-5p | 0.007 | 7.1e-04 | * |
| miR-5787 | 0.008 | 7.3e-04 | * |
| miR-194-5p | 0.011 | 8.0e-04 | * |
| miR-190a-5p | -0.014 | 8.1e-04 | * |
| miR-548am-5p | -0.005 | 8.3e-04 | * |
| miR-23a-3p | 0.009 | 8.4e-04 | * |
| miR-4685-5p | 0.011 | 9.2e-04 | * |
| let-7g-5p | -0.013 | 9.3e-04 | * |
| miR-15a-5p | -0.013 | 1.0e-03 | * |
| miR-550b-2-5p | -0.007 | 1.1e-03 | * |
| miR-144-3p | -0.028 | 1.2e-03 | * |
| miR-103a-2-5p | 0.010 | 1.2e-03 | * |
| miR-3124-3p | 0.005 | 1.3e-03 | * |
| miR-1273g-3p | 0.011 | 1.3e-03 | * |
| miR-497-3p | -0.004 | 1.5e-03 | * |
| miR-128-3p | 0.009 | 1.5e-03 | * |
| miR-6826-5p | 0.009 | 1.5e-03 | * |
| miR-31-3p | -0.004 | 1.6e-03 | * |
| let-7i-5p | -0.009 | 1.6e-03 | * |
| miR-885-5p | 0.005 | 1.7e-03 | * |
| miR-7846-3p | 0.010 | 1.7e-03 | * |
| miR-3928-3p | 0.004 | 1.8e-03 | * |
| miR-4513 | 0.010 | 1.8e-03 | * |
| miR-28-3p | 0.006 | 1.9e-03 | * |
| miR-4687-3p | 0.004 | 2.0e-03 | * |
| miR-550b-3p | 0.006 | 2.0e-03 | * |
| miR-501-3p | 0.006 | 2.0e-03 | * |
| miR-432-5p | -0.006 | 2.1e-03 | * |
| miR-339-3p | 0.008 | 2.1e-03 | * |

**Supplementary Table 14.** Differential expression analysis for 13 study-wide significant miRNAs identified in the meta-analysis by *Schulz J et al 2019*^1^ using Luxembourg Parkinson’s study miRNA dataset.

|  | miR-221-3p | miR-214-3p | miR-29c-3p | miR-29a-3p | miR-19b-3p | miR-193a-3p | **miR-141-3p**  **miR-451a** | miR-146a-5p | miR-133b | miR-15b-5p | **miR-185-5p**  miR-181a-5p |
| --- | --- | --- | --- | --- | --- | --- | --- | --- | --- | --- | --- |
| **HCvPD** | -0.11 | 0.03 | 0.07 | 0.05 | -0.07 | -0.04 | -**0.12*** -0.02**** | -0.07 | 0.08 | -0.05 **0.09**** 0.05 | |
| HCvPSP | -0.08 | -0.01 | 0.33 | 0.22 | -0.24 | 0.06 | -0.07 0.00 | 0.01 | 0.18 | 0.04 0.18 0.02 | |
| PDvPSP | 0.03 | -0.03 | 0.26 | 0.17 | -0.17 | 0.10 | 0.05 0.02 | 0.08 | 0.10 | 0.09 0.10 -0.03 | |
| HC_sex | 0.00 | 0.07 | 0.07 | -0.17** | 0.07 | 0.21** | 0.09 0.00 | -0.09 | -0.63*** | -0.04 -0.06 0.06 | |
| PD_sex | -0.11 | 0.04 | 0.16 | 0.03 | 0.02 | 0.03 | 0.02 0.01 | -0.06 | -0.49** | -0.02 0.11 -0.01 | |
| PSP_sex | 0.15 | -0.11 | 0.11 | 0.27 | -0.43 | -0.38 | -0.07 0.00 | 0.20 | -0.92 | -0.07 0.15 0.47 | |
| HC_AAA | -0.01** | 0.00 | 0.00 | 0.00 | 0.00 | 0.00 | 0.00** 0.00 | 0.00 | -0.01 | -0.01*** 0.00 0.00 | |
| PD_AAA | -0.01** | 0.00 | 0.01 | 0.01** | 0.01 | 0.01 | 0.00 0.00 | 0.00 | -0.01 | -0.01** 0.01** -0.01 | |
| PSP_AAA | -0.03 | 0.01 | 0.01 | 0.00 | 0.04 | 0.03 | 0.01 0.00 | -0.02 | 0.00 | 0.00 -0.01 -0.03 | |
| HCvPD_mlr | -0.02 | 0.01 | 0.01 | 0.00 | -0.10 | -0.06 | -0.09 -0.02** | -0.04 | 0.21 | 0.02 0.03 0.05 | |
| HCvPSP_mlr | 0.03 | -0.01 | 0.31 | 0.16 | -0.27 | 0.09 | -0.01 0.00 | 0.04 | 0.27 | 0.14 0.13 -0.02 | |
| PDvPSP_mlr | 0.06 | -0.04 | 0.23 | 0.13 | -0.20 | 0.07 | 0.06 0.01 | 0.09 | 0.06 | 0.11 0.08 0.01 | |
| HC_sex_mlr | 0.01 | 0.07 | 0.07 | -0.18** | 0.07 | 0.21** | 0.10 0.00 | -0.09 | -0.62*** | -0.03 -0.07 0.06 | |
| PD_sex_mlr | -0.09 | 0.04 | 0.14 | 0.01 | 0.01 | 0.03 | 0.02 0.01 | -0.05 | -0.48** | -0.01 0.09 0.00 | |
| PSP_sex_mlr | 0.13 | -0.11 | 0.11 | 0.27 | -0.41 | -0.36 | -0.07 0.00 | 0.19 | -0.93 | -0.08 0.14 0.45 | |
| HC_AAA_mlr | -0.01** | 0.00 | 0.00 | 0.00 | 0.00 | 0.00 | -0.01** 0.00 | 0.00 | -0.01 | -0.01*** 0.00 0.00 | |
| PD_AAA_mlr | -0.01** | 0.00 | 0.01 | 0.01** | 0.01 | 0.01 | 0.00 0.00 | 0.00 | -0.01 | -0.01** 0.01** -0.01 | |
| PSP_AAA_mlr | -0.03 | 0.01 | 0.01 | 0.00 | 0.03 | 0.03 | 0.01 0.00 | -0.02 | -0.01 | 0.00 -0.01 -0.03 | |

Significantly dysregulated miRNAs with the reported study-wide significant miRNAs in at least 3 independent datasets reported by *Schulz J et al 2019* are highlighted in bold (*miR-141-3p* and *miR-451a* overlapped in significance and direction and *miR-185-5p* overlapped in significance with various direction across the meta-analysed studies). Two or three asterisks mean significance corrected for multiple comparison using Benjamini-Hochberg of 5%, or a Bonferroni corrected 5% respectively. Significantly upregulated/downregulated miRNAs are highlighted in red/blue respectively. Abbreviations: microRNA (miR); control (HC); Parkinson’s disease (PD); progressive supranuclear palsy (PSP); multiple linear regression (mlr); age at assessment (AAA).

**Supplementary Table 15.** Target simulation for significantly dysregulated miRNAs in Parkinson’s disease (PD) vs. controls using Boolean modeling applied in the PD map (complete list including non-overlapping pathways (highlighted in *italics*) with PSP referred in the main manuscript).

| **Pathway** | **Dysregulated Molecules** | **Simulated Elements** | **Simulated Behaviour** | **Explanation** |
| --- | --- | --- | --- | --- |
| Transcription factor EB (TFEB) activity | MITF, PTEN, WDR45, RAB7A, PRKAG2, BECN1, ATP6VOD1, ATP6V1H, ATP6V1E1, ATP6V0E1, ATP6V1C1 | Lysosomal acidification  Autophagy | Impaired acidification  Compromised Autophagy | Alterations in TFEB activity affect autophagy and lysosomal function, crucial for the degradation of alpha-synuclein aggregates in PD. |
| Endoplasmatic reticulum (ER) stress signaling | CEBPB, DDIT4, MAP1LC3B, BECN1, HSP90B1 | Autophagy  Unfolded protein response (UPR)  Chaperone activity | Impaired Autophagy  Actived UPR  Modified chaperone activity | Chronic ER stress and the unfolded protein response contribute to the pathogenesis of PD by affecting protein folding and clearance mechanisms. |
| Calcium Signalling Pathway | ATP1A1, ATP1B1, SLC8A2, CACNA1D, CACNA2D2, CACNA2D3, CACNB2, ERN2, EIF2AK2, CAMK2, CAMK2B, PPP3CA, PPP3CB, YWHAB, RPS6KA1, RPS6KA3 | Calcium homeostasis  CREB signalling | Disrupted calcium homeostasis  Altered CREB signalling | Dysregulation of calcium signalling can lead to neuronal excitotoxicity and affect various signalling pathways implicated in PD. |
| Dopaminergic Transcription | NR4A2, NCOR2, FOXA2, SNCA, PARK7(DJ1), CGH1, TH, DDC, SLC18A, ALC6A3, CFLAR, SOD1, COX5B, COX6A1, NDUFB8, BDNF, RET, ALDH1A1, FOXO1, FOXO3, KLC1, MAP1B, EN1, FOXA1, FOXA2 | Transcriptional regulation  Mitochondrial function | Dysregulated transcription  Compromised mitochondrial function | Impairment in the transcriptional machinery and mitochondrial dysfunction are key features in the loss of dopaminergic neurons in PD. |
| PPARGC1A Activity | SIRT1, ESR, IDH3B, IDH3G, COX5B, COX6A, COX6B1, COX7A2, COX7C, SDHB, TOMM20, SURF1, VDAC1, TFB2M | Mitochondrial biogenesis  Respiratory function | Altered biogenesis  Impaired respiratory function | PPARGC1A is a key regulator of mitochondrial biogenesis and function, and its dysregulation is implicated in the mitochondrial dysfunction observed in PD.  Linked to diabetes/insulin resistance. |
| *Actin Filament* | ACTR3, ALDRISH PROTEIN, CAPNS1, CDC42, CDK5R1, CFL1, CTNND2, OMG, PAK6_phosphorylated, PIN1, PFN2, PRDX2, RAC1, RTN4, RDX, SNCA | Actin filament organization  Neurite outgrowth  Spine growth | Disruption in actin filament organization  Impaired neurite outgrowth  Reduced spine growth | Alterations in actin filament-associated molecules lead to cytoskeletal instability, affecting neuronal morphology, synaptic connectivity, and signal transmission. |
| *Axonal Guidance* | CDK5R1, CTNND2, PIN1, PRDX2, SNCA, CAPNS1, RTN4, OMG, CFL1, PFN2, ACTR3, WAS, WASF1, PAK6, RDX, CDC42 | Neurite outgrowth  Dendritic outgrowth  Spine outgrowth | Impaired neurite and dendritic outgrowth  Reduced spine outgrowth | Dysregulation in these molecules leads to defective axonal guidance, affecting neuronal connectivity and plasticity. |
| *MTOR Signalling* | DDIT4, PHLPP1, SIRT1 | AKT activation  catabolism  Glycolysis | Altered AKT activation  Disrupted catabolism and glycolysis | Changes in mTOR signalling affect cellular metabolism and survival, impacting neuronal health. |
| *PRKN Mitophagy* | ATXN3, DNM1L, HSPA1L, HSPA9, NEDD8, OPTN, PARL, PMPCA, PMPCB, VPS13C | Mitophagy  PINK1  PRKN | Impaired mitophagy  Altered PINK1/PRKN activity | Dysregulation in these molecules affects the process of mitophagy, essential for mitochondrial quality control in neurons. |

Column *dysregulated molecules* refers to the target molecules within the dysregulated pathway(s) in the simulation. Column *simulated elements* reflects on the broader impact at the cellular or system level highlighting the elements that are simulated or affected due to the molecular dysregulation. Column *simulated behaviour* describes the consequent aberrant behaviour or states induced by the pathway dysregulation(s).

**Supplementary References**

^1^Schulz J, Takousis P, Wohlers I, et al. Meta‐analyses identify differentially expressed microRNAs in Parkinson’s disease. *Ann Neurol*. 2019;85(6):835-851. doi:10.1002/ana.25490
